# EAE of Mice: Enzymatic Cross Site-Specific Hydrolysis of H2B Histone by IgGs against H1, H2A, H2B, H3, and H4 Histones and Myelin Basic Protein

**DOI:** 10.3390/molecules28072973

**Published:** 2023-03-27

**Authors:** Andrey E. Urusov, Kseniya S. Aulova, Pavel S. Dmitrenok, Valentina N. Buneva, Georgy A. Nevinsky

**Affiliations:** 1 Institute of Chemical Biology and Fundamental Medicine, Siberian Division of Russian Academy of Sciences, Lavrentiev Ave. 8, 630090 Novosibirsk, Russia; 2 G. B. Elyakov Pacific Institute of Bioorganic Chemistry, Far East Division, Russian Academy of Sciences, 690022 Vladivostok, Russia

**Keywords:** C57BL/6 mice, EAE model of human multiple sclerosis, immunization mice with MOG and DNA-histone complex, catalytic antibodies, hydrolysis of histones and myelin basic protein, cross-complexation and catalytic cross-reactivity

## Abstract

Histones have vital roles in chromatin functioning and gene transcription. At the same time, they are pernicious in intercellular space because they stimulate systemic inflammatory and toxic responses. Myelin basic protein (MBP) is the major protein of the axon myelin–proteolipid sheath. Antibody-abzymes with various catalytic activities are specific features of some autoimmune diseases. IgGs against five individual histones (H2B, H1, H2A, H3, and H4) and MBP were isolated from the blood of experimental autoimmune encephalomyelitis-prone C57BL/6 mice by affinity chromatography. Abzymes corresponding to various stages of EAE development, including spontaneous EAE, myelin oligodendrocyte glycoprotein (MOG)- and DNA-histone complex-accelerated onset, as well as acute and remission stages, were analyzed. IgG-abzymes against MBP and five individual histones showed unusual polyreactivity in complex formation and enzymatic cross-reactivity in the specific hydrolysis of H2B histone. All IgGs against MBP and individual histones in 3-month-old mice (zero time) demonstrated from 4 to 11 different H2B hydrolysis sites. Spontaneous development of EAE during 60 days led to a significant change in the type and number of H2B hydrolysis sites by IgGs against the five histones and MBP. Mouse treatment with MOG and DNA-histone complex changed the type and number of H2B hydrolysis sites compared to zero time. The minimum number (3) of different H2B hydrolysis sites was found for IgGs against H3 20 days after mouse immunization with DNA-histone complex, whereas the maximum number (33) for anti-H2B IgGs was found 60 days after mouse treatment with DNA-histone complex. Overall, this is the first study to demonstrate that at different stages of EAE evolution, IgG-abzymes against five individual histones and MBP could significantly differ in the specific sites and number of H2B hydrolysis sites. Possible reasons for the catalytic cross-reactivity and significant differences in the number and type of histone H2B cleavage sites were analyzed.

## 1. Introduction

Histones and their various modified forms play a vital role in chromatin functioning. Free extracellular histones usually act as damage factors [1]. Treatment of experimental mice with five exogenous histones led to systemic toxic responses, including inflammatory reactions and activation of Toll-like receptors [1]. Treatment of mice with antibodies (Abs) neutralizing five histones, heparin, activated protein C, and thrombomodulin provided mice protection against sepsis, trauma, lethal endotoxemia, ischemia–reperfusion injury, pancreatitis, stroke, peritonitis, coagulation, and thrombosis. The increased levels in the blood of free histones and nucleosome fragments cause several pathophysiological processes, including progression in inflammatory processes, several autoimmune diseases (AIDs), and cancer [1].

The core tetramers of nucleosome particles contain two molecules of H4 and H3, and they are circled by two dimers of histones H2A and H2B bound with two supercoiled turns of double-stranded DNA [2]. H1 histone is essential for packing chromatin and forming a higher-order chromatin structure. H2B histone plays a vital role in nuclear biology, where it is involved in packaging and maintaining chromosomes, regulating transcription, and DNA replication and repair. Histone H2B is important for the regulation of chromatin structure and function through post-translational modifications.

In several different AIDs, including multiple sclerosis (MS) and systemic lupus erythematosus (SLE), antibodies to DNA and histones are primarily directed against DNA-histone nucleosomal complexes emerging in the blood due to the apoptosis of cells [3].

MS is an inflammatory demyelinating AID of the central nervous system. Many macrophages characterize this pathology as well as T lymphocytes in the perivascular infiltrate [4]. Some data attesting to MS pathogenesis include the essential role of B cells and auto-Abs against myelin autoantigens, including myelin basic protein (MBP) [4,5,6,7]. The activated myelin-reactive CD4+ T cells could be the principal mediators of multiple sclerosis [4].

Several experimental autoimmune encephalomyelitis (EAE) mice models mimic well the peculiar properties of human MS (for review, see ref. [8,9]). Autoimmune diseases were first proposed to originate from particular defects of bone marrow hematopoietic stem cells (HSCs) [9]. Later, it was demonstrated that the spontaneous and antigen-induced evolution of autoimmune diseases develops from a specific autoimmune reorganization of bone marrow HSCs [10,11,12,13,14,15,16]. C57BL/6 mice prone to EAE were previously used for the investigation of possible mechanisms of spontaneous and myelin oligodendrocyte glycoprotein (MOG) as well as DNA–protein complexes [11,12,13,14] acceleration of EAE development. The studies showed that treatment of SLE-prone MRL-lpr/lpr mice with DNA–protein complex [15,16,17] and C57BL/6 mice with DNA-histone complex or MOG [11,12,13,14] procured a significant speed-up of SLE and EAE development. Such acceleration was a consequence of specific changes in the HSC differentiation profile and a significant increase in lymphocyte proliferation, thus producing abzymes in various organs of mice [11,12,13,14,15,16,17].

Moreover, these changes in the differentiation profile were associated with the production of various auto-Abs-abzymes (Abzs) that hydrolyze DNA, polysaccharides, RNA, proteins, and peptides. The detection of different auto-abzymes is the earliest and statistically significant marker of the beginning and progress of many AIDs [11,12,13,14,15,16,17,18,19,20,21,22,23,24]. Catalytic activities of Abzs are easily detectable even at the onset of several AIDs (at the pre-disease stage corresponding to the beginning of AIDs) before the discovery of typical markers of different autoimmune pathologies [11,12,13,14,15,16,17,18,19,20,21,22,23,24]. Titers of auto-Abs to peculiar auto-antigens at the onset of many AIDs usually correspond to standard index ranges in the blood of healthy humans and mice [19,20,21,22,23]. The appearance of multiple Abzs clearly demonstrates the start of autoimmune reactions when an incremental increase in the catalytic activity of antibodies is related to the development of profound pathologies. However, several parallel mechanisms could cause the development of different autoimmune pathologies, eventually resulting in a breakdown of self-tolerance [19,20,21,22,23].

Natural auto-abzyme splitting of various oligosaccharides, proteins, peptides, nucleotides, DNA, and RNA was detected in the blood of patients with several AIDs and viral diseases [18,19,20,21,22,23]. Auto-abzymes with shallow activities hydrolyzing polysaccharides [24], thyroglobulin [25], and vasoactive neuropeptide [26,27] were found in the blood of some conditionally healthy volunteers. However, conditionally healthy humans and animals usually lack Abzs [18,19,20,21,22,23]. Nevertheless, some germline auto-antibodies of healthy people could possess detectable levels of some amyloid-, superantigen-, and microbe-directed activities [28,29].

Similar to SLE patients [23], the blood plasma of MS patients contains abzymes splitting DNA and RNA [30,31,32], MBP [33,34,35,36], oligosaccharides [19,24], and histones [37]. The relative activities (RAs) of IgG-Abzs from the cerebrospinal fluids of patients with MS degrading MBP, polysaccharides, and DNA are, on average, 30–60 times higher than those from the blood of the same patients [38,39,40]. In various AIDs, abzymes against MBP can hydrolyze this protein in the myelin–proteolipid sheath of axons. They may, therefore, play a very adverse role in the pathogenesis of MS, SLE, and other AIDs [18,19,20,21,22,23].

DNA-histone complexes are the main auto-antigens in producing Abs and abzymes against DNA and histones [4], which are very toxic for mammals. Abs-abzymes splitting five histones (H1, H2A, H2B, H3, and H4) are found in the sera of MS [37] and HIV-infected patients [41,42,43,44,45,46] and in EAE mice [11,47]. As mentioned above, five free extracellular histones act as damage molecules [1]. These abzymes can penetrate through membranes of cells and nuclei, hydrolyze chromatin DNA, and induce the apoptosis of cells [48,49,50]. Therefore, Abzs splitting MBP, DNA, and histones may be essential in the pathogenesis of MS and other AIDs.

It is believed that the development of different AIDs could be associated with human infection by various bacteria and/or viruses, including human herpesvirus, human endogenous retroviruses, and Epstein–Barr virus (for review, see ref. [51,52,53,54,55,56]). At first, there might be the production of Abs against bacterial or viral compounds that possess a high level of homology with human proteins [51,52,53,54,55,56]. Later, due to the strong mimicry of some viral or bacterial proteins with those of humans, immune system violations could result in the generation of auto-Abs to human proteins and the evolution of AIDs. The treatment { XE “immunzation” } of different autoimmune-prone mice { XE “mice” } with different antigens led to a significantly higher incidence { XE “incidence” } of abzyme production with higher enzymatic activities than in normal { XE “normal” } conventionally used mouse { XE “mouse” } strains { XE “strains” } [57,58].

The unspecific complex formation of various proteins and enzymes with foreign ligands is described as a widespread phenomenon [59,60,61]. The efficiency of the correct selection of specific substrates by enzymes during complex formation usually does not exceed 1–2 orders of magnitude [59,60,61]. It is the subsequent specific changes in enzymes and substrate structures that lead to the catalysis stage and increase the reaction rate by 5–8 orders of magnitude for specific versus non-specific substrates [59,60,61]. Therefore, enzymatic cross-reactivity regarding substrates in the case of canonical enzymes is very sporadic [59,60,61]. Typically, classical enzymes usually catalyze only one chemical reaction.

Non-specific complex formation of some proteins, nucleic acids, and other ligands with antibodies against other ones detectable by ELISA or affinity chromatography is a widespread phenomenon known as Abs polyspecificity or polyreactivity complexation [62,63,64,65]. Abzymes against many different proteins, similar to classical enzymes, usually specifically hydrolyze only one specific antigen protein and cannot split many other control unspecific globular proteins ([18,19,20,21,22,23] and refs therein). First, research showed that anti-MBP Abzs hydrolyzed only MBP [33,34,35,36,37] while abzymes against histones only hydrolyzed histones [41,42,43,44]. However, analysis of abzymes has shown that the immune response to auto-antigens in AIDs is much more complex and multifaceted than could be seen based on classical immunology.

The catalytic cross-activity of any Abs-Abzs against various proteins was recently described [18,19,20,21,22,23]. It was shown that polyclonal auto-IgGs of HIV-infected patients against MBP specifically hydrolyzed MBP and five H1-H4 histones and vice versa—total preparations of abzymes against the five histones effectively degraded MBP [45,46]. Recently, IgGs against five histones and MBP corresponding to different stages of EAE development, including spontaneous EAE, MOG- and DNA-histone complex-accelerated onset, acute, and remission stages in C57BL/6 mice were analyzed [47]. Such IgG-abzymes against five histones and MBP demonstrated unusual polyreactivity in complex formation and enzymatic cross-reactivity in the hydrolysis of H4 histone. These chimeric abzymes with cross-catalytic reactivity could be very hazardous for the development of many AIDs since Abzs against five histones can also hydrolyze MBP of nerve tissue shells [45,46]. It seemed interesting to what extent the phenomenon of enzymatic cross-reactivity between Abzs against histones and MBP is common in humans and animals with different AIDs. In addition, it was important to understand whether there is unusual enzymatic cross-reactivity of Abs-Abzs against histones and MBP only in the case of H4 or also for other histones. Moreover, the analysis of abzymes corresponding to different stages of EAE development in C57BL/6 mice can allow an understanding of how the relative activities of abzymes and their substrate specificity concerning individual histones and MBP can change depending on the stage of pathology.

Here, we showed that abzymes of mice against each of the five individual histones and MBP possessed catalytic cross-reactivity in splitting H2B histone. Moreover, it was demonstrated that abzymes against each of the five histones and MBP corresponding to different stages of EAE development could hydrolyze H2B histone with different efficiencies and in various specific sites. Therefore, this study is the first to analyze the hydrolysis of H2B with IgGs against five individual H1-H4 histones and MBP corresponding to different stages of EAE development.

## 2. Results

### 2.1. Choosing a Model for Catalytic Cross-Reactivity Analysis

The human immune system could theoretically produce about a million variants of antibodies having different properties against one individual antigen [66]. The possibility of studying a possible diversity of antibodies against external and internal specific antigens using enzyme-linked immunosorbent assay (ELISA) and affinity chromatography is very limited.

Unlike antibodies without activity, abzymes differ not only in their affinity for the same antigen substrate but also in the rate of hydrolysis, optimal pH, dependence or independence from various metal ions, possible cofactors, etc. It was demonstrated that only the analysis of abzymes could help to elicit an exceptionally expanded diversity of antibodies against the same antigens (for review, see ref. [18,19,20,21,22,23]). An approximate evaluation of the possible diversity of antibodies, for example abzymes, in terms of their different properties was carried out in several articles [67,68,69,70,71,72,73]. A cDNA library of κ-type light chains of antibodies from SLE patients and a phage display method were used to obtain individual monoclonal light chains (MLChs) of Abs. The pool of phage particles containing various antibodies on the surface was divided into ten peaks eluted from MBP-Sepharose using different NaCl concentrations [67,68,69,70,71]. MLChs bound to phage particles corresponding to 10 peaks eluted from affinity sorbent effectively hydrolyzed MBP. Phage particles of one peak eluted at 0.5 M NaCl were used to obtain individual colonies and then isolate MLChs [67,68,69,70,71,72,73]. MLChs corresponding to 72 preparations haphazardly selected from 440 individual colonies were analyzed, and 22 of 72 preparations (~30%) possessed MBP-hydrolyzing activity. Of the 22 MLChs with a comparable affinity for MBP-Sepharose (one peak), 12 possessed metal-dependent activity, 4 had serine-like activity, and 3 possessed thiol protease activity. Two MLChs demonstrated both serine-like and metalloprotease activities combined in one active site, and one had three activities—serine-like, metalloprotease, and DNase activities [67,68,69,70,71,72,73].

Unlike other different methods of antibody analysis, the analysis of the optimal conditions for the manifestation of catalytic activity by antibodies allows us to reveal the exceptional diversity of antibodies with comparable affinity for the same antigen. Such analysis showed that all MLCh–abzyme preparations differed significantly in relative activity, optimal concentrations of different metal ions (Na^+^, K^+^, Mg^2+^, Zn^2+^, Mn^2+^, Co^2+^, Ni^2+^, etc.), pH optima, and other properties [67,68,69,70,71,72,73].

Analysis of MLCh protein sequence homology with that of known classical Zn^2+^- and Ca^2+^-dependent as well as human serine-like and thiol proteases was carried out. The DNA sequences of these MLChs were appropriate (88–100%) to the germ lines of the IgLV8 light chain genes of several described antibodies [71,72,73]. The MLCh protein sequences responsible for binding MBP, metal ion chelation, and direct catalysis turned out to be very close to those for canonical proteases [71,72,73].

It should be emphasized that antibodies of all peaks eluted from MBP-Sepharose possessed MBP-hydrolyzing activities. If we take into account the average value of the percentage of active abzymes in one peak, which was approximately 30%, the minimum peak value of 10, as well as the number of individual colonies analyzed, then the possible number of only κ–type Abzs with MBP-hydrolyzing activity could be ≥1000. However, MBP-hydrolyzing activity was shown for Abs possessing kappa and lambda chains [20,21,22,23]. These data testify to the extreme diversity of abzymes to the same antigen. In addition, it was evident that in the same active center of abzymes, unlike in classical enzymes, amino acid residues responsible for the manifestation of several different catalytic functions could be combined.

The evolution of EAE in C57BL/6 mice occurs spontaneously. Treating mice with DNA-histone complex [11,12] or MOG can significantly accelerate EAE development [13,14]. There are three main stages of EAE progress after mouse immunization with antigens: onset at 7–8 days, the acute stage at 18–20 days, and the remission phase later at 25–30 days. The acceleration of EAE achievement is associated with specific changes in the bone marrow HSC differentiation profile and a rise in lymphocyte proliferation [11,12,13,14]. These processes stimulate the production of lymphocytes synthesizing Abzs, splitting DNA, RNA, MBP, MOG, and histones. The parameters characterizing all of these specific changes in mice were previously analyzed [11,12,13,14]. To assay the enzymatic cross-reactivity of IgGs in the hydrolysis of H2B histone, we chose two antigens described earlier: MOG [13,14] and DNA-histone complex [11,12]. Data showing the changes in the differentiation profile of HSCs before and after mouse immunization with MOG and DNA-histone complex are presented in the Supplementary Data (Supplementary Figure S1). The changes in the relative concentrations of different antibodies are given in Supplementary Figure S2. The changes in the relative activities (RAs) of IgG-abzymes in the hydrolysis of MOG, MBP, DNA, and histones during EAE development are given in Supplementary Figure S3. One could see that during the development of spontaneous EAE, the rise in the relative amounts of four precursors of hemopoietic cells (CFU-E, CFU-GM, BFU-E, and CFU-GEMM) in the bone marrow of C57BL/6 mice was relatively gradual and slow. Mouse treatment with MOG and DNA-histone complex resulted in various changes over time in the profile of stem cell differentiation, but EAE development was accelerated in all cases.

Previously, catalytic cross-reactivity was shown using the example of total antibodies against five histones from the blood of HIV-infected patients and IgGs against MBP [45,46]. Recently, we analyzed the possible enzymatic cross-reactivity of total IgGs of C57BL/6 mice against five histones (isolated using Sepharose containing five immobilized histones) and MBP in the hydrolysis of H4 histone [47]. The results showed that the treatment of mice with MOG and DNA-histone complex resulted in the hydrolysis of H4 histones at different sites. In addition, the sites of H4 histone hydrolysis by IgGs against the five histones and MBP significantly changed depending on the stage of development of EAE, including onset, the acute phase, and remission stage.

In this work, antibodies against five individual histones were isolated for the first time from preparations of total antibodies against five histones and a study of their catalytic cross-activity in the hydrolysis of MBP and H2B histone was performed.

### 2.2. Purification of Antibodies

Purification of electrophoretically homogeneous IgG preparations (IgG_mix_; mixture of 7 plasma blood samples) corresponding to each of the mice groups using Protein G-Sepharose and FPLC gel filtration under drastic conditions (pH 2.6) was performed as previously described [47]. After SDS-PAGE of the IgG_mix_ preparations, protease activity in the hydrolysis of histones and MBP was detected only in one 150 kDa IgG band, and there were no other proteins or protease activity peaks (Supplementary Figure S4). This indicated that the IgG_mix_ preparations corresponding to each of the mice groups did not contain impurities of canonical proteases having molecular weights of 20–40 kDa.

IgGs against five histones corresponding to each of the mice groups were previously purified by chromatography on histone5H-Sepharose containing five immobilized histones [47]. Non-specifically bound IgG fractions and those having low affinity for the five histones were first eluted with 0.2 M NaCl. Anti-histone-specific IgGs having a high affinity for the five immobilized histones were then eluted with Tris-Gly buffer, pH 2.6 as in ref. [47]. For additional purification of IgGs against the five histones from potential impurities of IgGs against MBP, the fraction from histone5H-Sepharose was passed through MBP-Sepharose. The fraction obtained after loading onto MBP-Sepharose was further used as the anti-5-histones IgGs for purification of IgGs against each of the five individual histones.

The following 35 IgG preparations against all five histones, individual histones, and MBP corresponding to different groups of mice and different stages of EAE development were analyzed (Table 1).

### 2.3. SDS-PAGE Analysis of Enzymatic Cross-Reactivity

**Figure 1:**
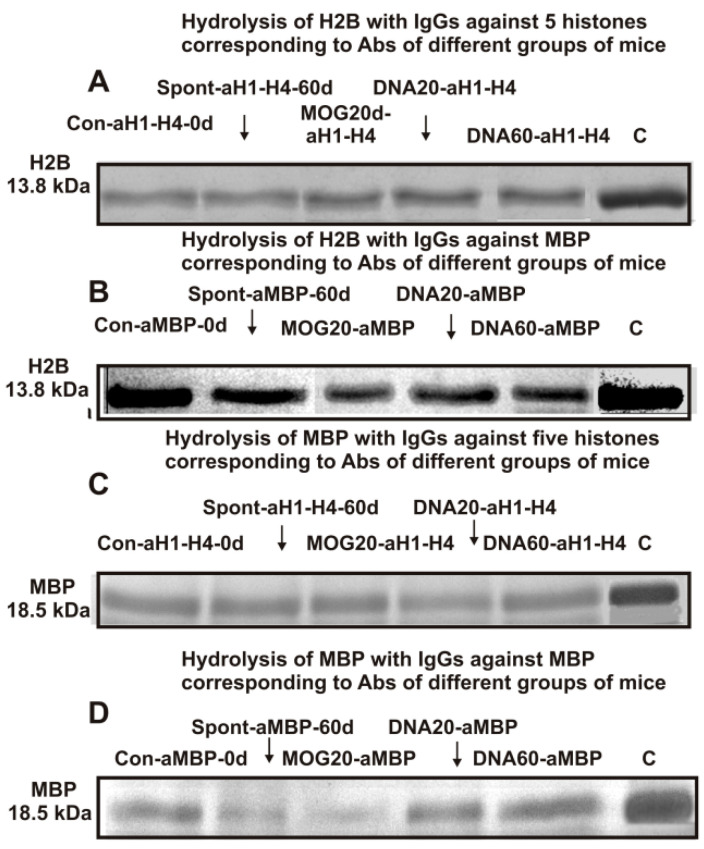
SDS-PAGE analysis of H2B (13.8 kDa) hydrolysis by IgGs against the five histones (**A**) and Abs against MBP (**B**) as well as splitting myelin basic protein (18.5 kDa) by antibodies against the five histones (**C**) and IgG-abzymes against MBP (**D**). Lanes C correspond to the histones (**A**,**B**) and MBP (**C**,**D**) incubated without IgG-abzymes. MBP and a mixture of five histones with and without IgGs (0.03 mg/mL) were incubated for 14 h. The full gel pictures are given in Supplementary Data (addition to Figure 1).

The possible enzymatic cross-reactivity of anti-5-histones IgGs (eluted from histone5-Sepharose) and anti-MBP IgGs (eluted from MBP-Sepharose) was first analyzed. Figure 1A,B demonstrate the hydrolysis of H2B histone with anti-5-histones IgGs and anti-MBP IgGs, while Figure 1C,D show the hydrolysis of MBP by these IgGs.

The efficiencies of H2B and MBP cleavage with various IgGs (Figure 1) were calculated from the decrease in the amount of proteins in the initial bands after incubation with IgG-abzymes compared to their content after incubation without Abs (lanes C, Figure 1). After 14 h of H2B incubation with Abs against MBP and histones, the relative content of H2B (~13.8 kDa) and MBP (18.5 kDa) decreased remarkably and or significantly compared to the control experiment (lanes C). As is known, catalysis of the reactions of enzymes and abzymes occurs after the formation of their complexes with substrates. Therefore, these data suggested that the anti-histones and anti-MBP IgGs of mice possessed a previously discovered phenomenon of different Abs having semi-specific complex formation polyreactivity [62,63,64,65] and enzymatic cross-reactivity in MBP and histone hydrolysis [45,46]. These data, however, could not guarantee absolute proof of enzymatic cross-reactivity between IgGs against MBP and the five histones because it could not rule out that the obtained preparations, notwithstanding, could contain very small admixtures of IgGs against alternative histones or MBP even after isolation of the IgGs by affinity chromatography. Stronger proof of enzymatic cross-reactivity may be obtained from a significant difference in the specific sites of H2B histone hydrolysis by IgGs against MBP and five individual histones. This study first analyzed the possibility of hydrolysis of histone H2B with specific IgG-abzymes against the five individual histones and MBP.

### 2.4. MALDI Spectra of H2B Histone Hydrolysis

As shown using IgG-abzymes of HIV-infected patients against five histones, they split all histones and MBP and vice versa [45,46]. IgGs of C57BL/6 mice also hydrolyzed all five histones and MBP [11,12,13,14]. Moreover, IgG-abzymes against MBP and five histones of C57BL/6 mice demonstrated cross-enzymatic activity in the splitting of H4 histone [47]. The sites of H4 hydrolysis by a set of different IgGs depended on their type and the stage of EAE development.

**Figure 2:**
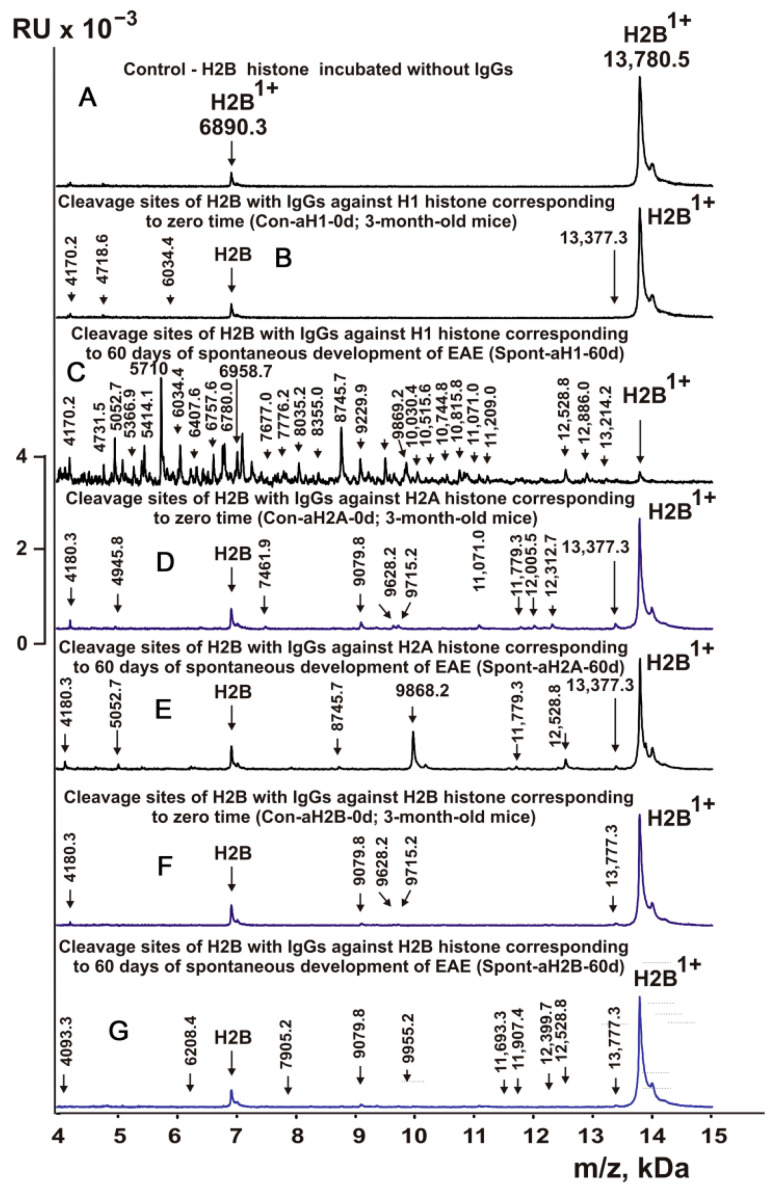
MALDI spectra corresponding to products of H2B histone (0.9 mg/mL) hydrolysis in the absence (**A**) or presence of IgGs (0.04 mg/mL) against three histones: control (H2B incubated without Abs) (**A**), Con-aH1-0d (**B**), Spont-aH1-60d (**C**), Con-aH2A-0d (**D**), Spont-aH2A-60d (**E**), Con-aH2B-0d (**F**), and Spont-aHB-60d (**G**). All designations of IgG preparations and values of *m*/*z* are shown in the figure.

In this work, IgGs from mice corresponding to individual histones and MBP and corresponding to different stages of EAE development (Table 1) were first used to study their enzymatic cross-reactivity in the hydrolysis of H2B histone. The cleavage sites of H2B were identified by MALDI mass spectrometry. After incubation in the absence of IgGs (Figure 2A), H2B histone was almost homogeneous, showing two signals of its one- (*m*/*z* = 13,780.5 Da) and two-charged ions (*m*/*z* = 6990.3 Da). First, H2B hydrolysis sites were identified using IgGs against three individual histones corresponding to the zero time of the experiment (3-month-old mice) and the spontaneous development of EAE during 60 days (without mouse immunization). For all IgG preparations against individual histones listed in Table 1, 8–10 spectra were obtained. Several typical spectra are shown in Figure 2. Each preparation of IgGs demonstrated a specific set of peaks corresponding to the hydrolysis of H2B histone.

**Figure 3:**
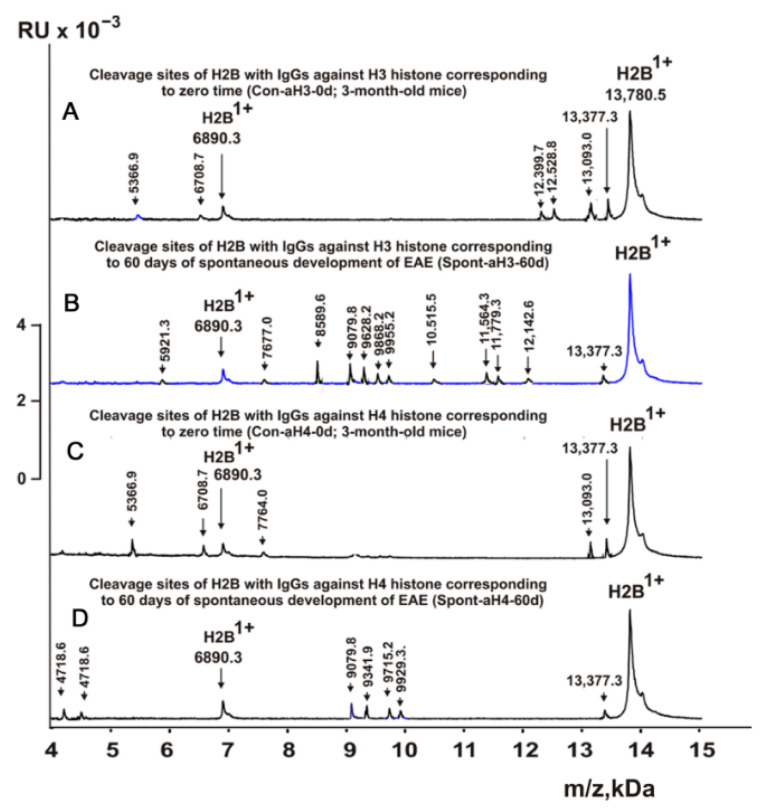
MALDI spectra corresponding to products of H2B histone (0.9 mg/mL) hydrolysis in the presence of IgGs (0.04 mg/mL) against two histones corresponding to zero time and after spontaneous development of EAE during 60 days: Con-aH3-0d (**A**), Spont-aH3-60d (**B**), Con-aH4-0d (**C**), and Spont-aH4-60d (**D**). All designations of IgG preparations and values of *m*/*z* are shown in the figure.

**Figure 4:**
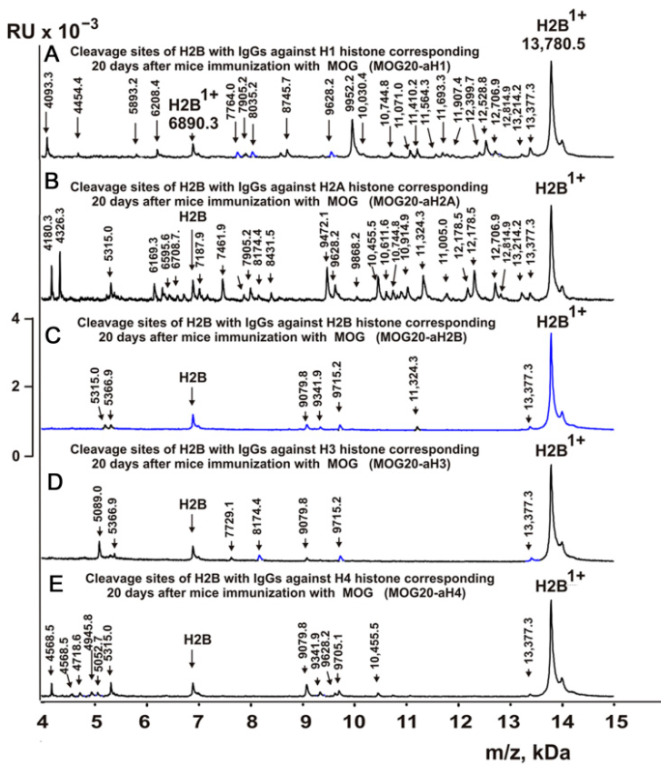
MALDI spectra of H2B (0.9 mg/mL) hydrolysis products with IgGs against the five histones corresponding to 20 days after mouse immunization with MOG: MOG20-aH1 (**A**), MOG20-aH2A (**B**), MOG20-aH2B (**C**), MOG20-aH3 (**D**), and MOG20-aH4 (**E**). All designations of IgG preparations and values of *m*/*z* are shown in the figure.

**Figure 5:**
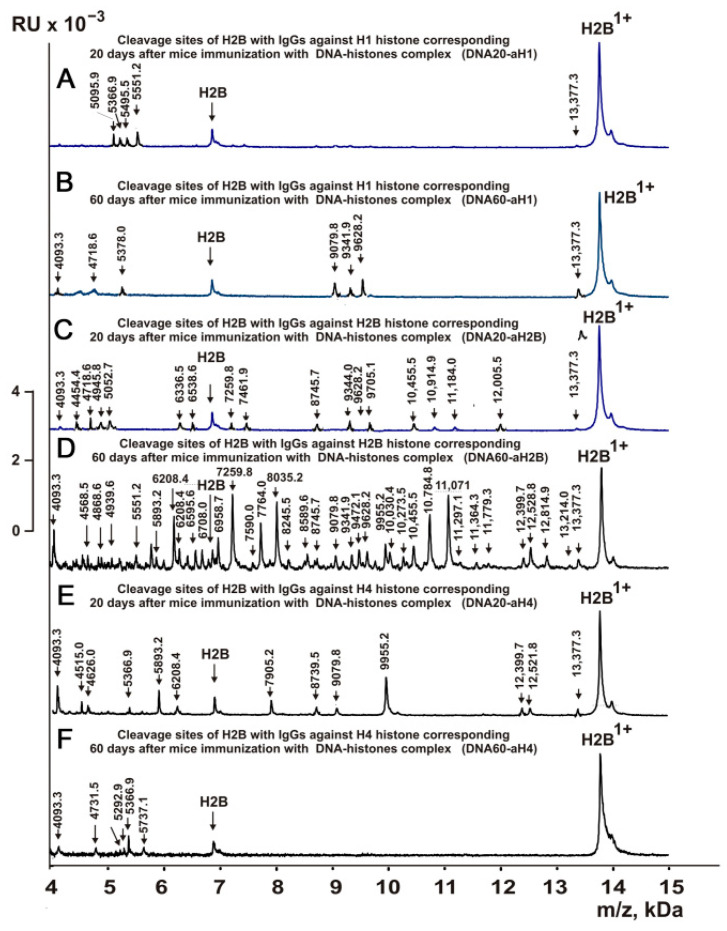
MALDI spectra of H2B (0.9 mg/mL) hydrolysis products with IgGs against three histones corresponding to 20 and 60 days after mouse immunization with DNA-histone complex: DNA20-aH1 (**A**), DNA60-aH1 (**B**), DNA20-aH2A (**C**), DNA60-aH2A (**D**), DNA20-aH2B (**E**), and DNA60-aH2B (**F**). All designations of IgG preparations and values of *m*/*z* are shown in the figure.

**Figure 6:**
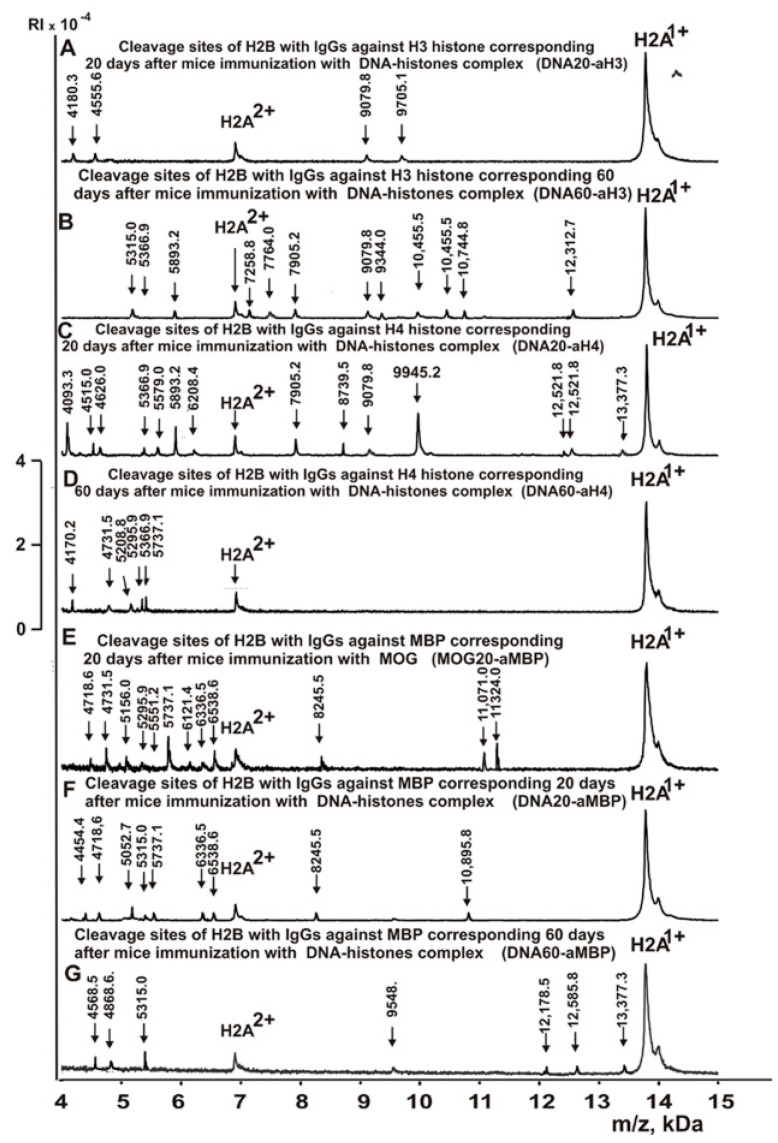
MALDI spectra of H2B (0.9 mg/mL) hydrolysis products with IgGs against two histones and MBP corresponding to 20 and 60 days after mouse immunization with DNA-histone complex: DNA20-aH3 (**A**), DNA60-aH3 (**B**), DNA20-aH4 (**C**), DNA60-aH4 (D), MOG20-aMBP (**E**), DNA20-aMBP (**F**), and DNA60-aMBP (**G**). All designations of IgG preparations and the values of *m*/*z* are shown in the Figure.

Figure 3, Figure 4, Figure 5 and Figure 6 demonstrate MALDI spectra corresponding to the hydrolysis of H2B by IgGs against various histones and MBP after immunization of mice with MOG and DNA-histone complex.

### 2.5. Sites of H2B Histone Hydrolysis

As can be seen from Figure 2, Figure 3, Figure 4, Figure 5 and Figure 6, each IgG preparation demonstrated its own specific set of peaks corresponding to H2B hydrolysis products with different molecular weights. All sites of H2B histone hydrolysis by all IgGs against individual histones and MBP are summarized in Figure 7, Figure 8, Figure 9, Figure 10 and Figure 11.

Some data on the sites of H2B hydrolysis by IgGs against H3 and H4 histones are shown in Figure 8.

**Figure 7:**
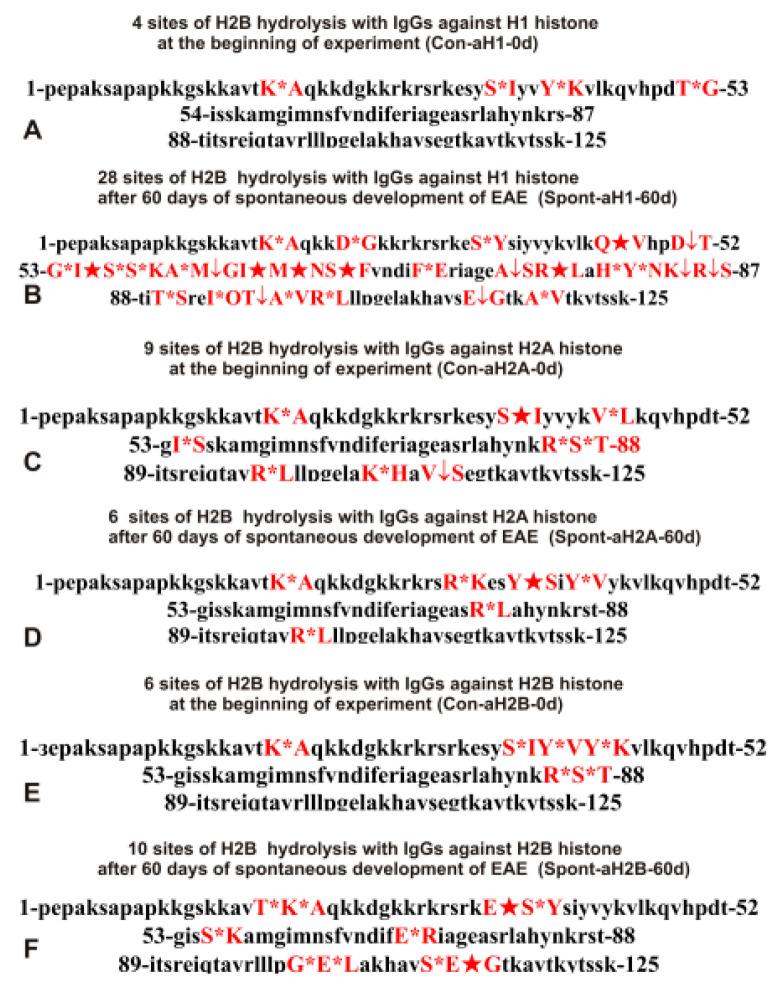
Sites of H2B hydrolysis by IgGs against three histones (H1, H2A, and H2B) corresponding to zero time (3-month-old mice) and after spontaneous development of EAE (before mouse immunization) during 60 days: Con-aH1-0d (**A**), Spont-aH1-60d (**B**), Con-aH2A-0d (**C**), Spont-aH2A-60d (**D**), Con-aH2B-0d (**E**), and Spont-aH2B-60d (**F**). Major sites of H2B cleavage are shown by stars (★), moderate sites by arrows (↓), and minor sites of cleavage by small stars (*).

**Figure 8:**
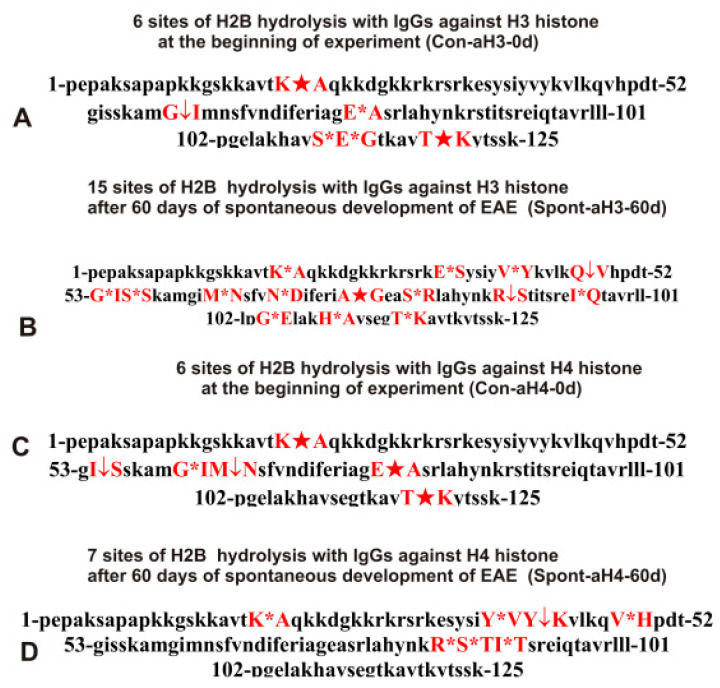
Sites of H2B hydrolysis by IgGs against H3 and H4 histones corresponding to zero time (3-month-old mice) and after spontaneous development of EAE (before mouse immunization) during 60 days: Con-aH3-0d (**A**), Spont-aH3-60d (**B**), Con-aH4-0d (**C**), and Spont-aH4-60d (**D**). Major sites of H2B cleavage are shown by stars (★), moderate sites by arrows (↓), and minor sites of cleavage by small stars (*).

**Figure 9:**
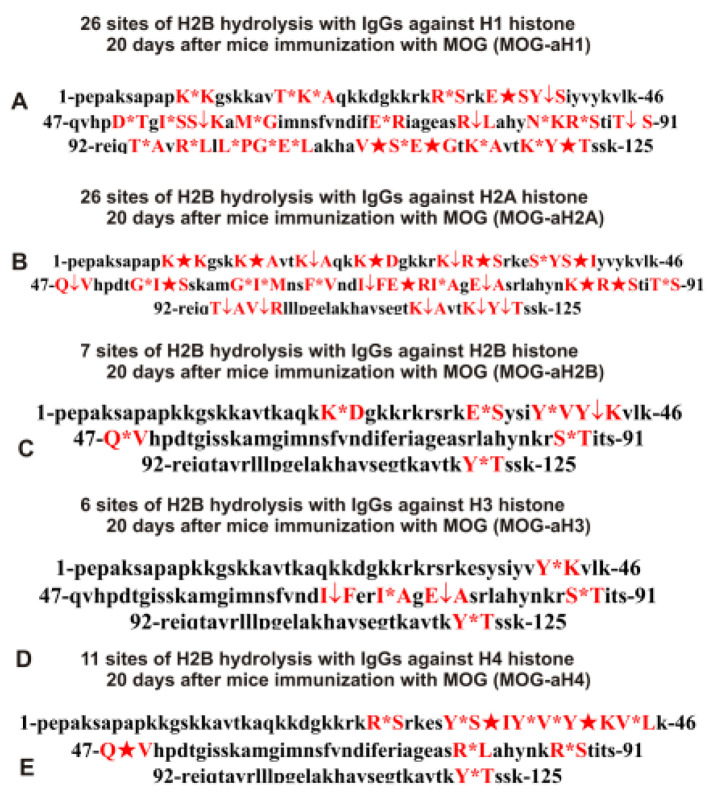
Sites of H2B hydrolysis by IgGs against five H1-H4 histones corresponding to 20 days after mouse immunization with MOG: MOG20-aH1 (**A**), MOG20-aH2A (**B**), MOG20-aH2B (**C**), MOG20-aH3 (**D**), and MOG20-aH4 (**E**). Major sites of H1 cleavage are shown by stars (★), moderate sites by arrows (↓), and minor sites of cleavage by small stars (*).

**Figure 10:**
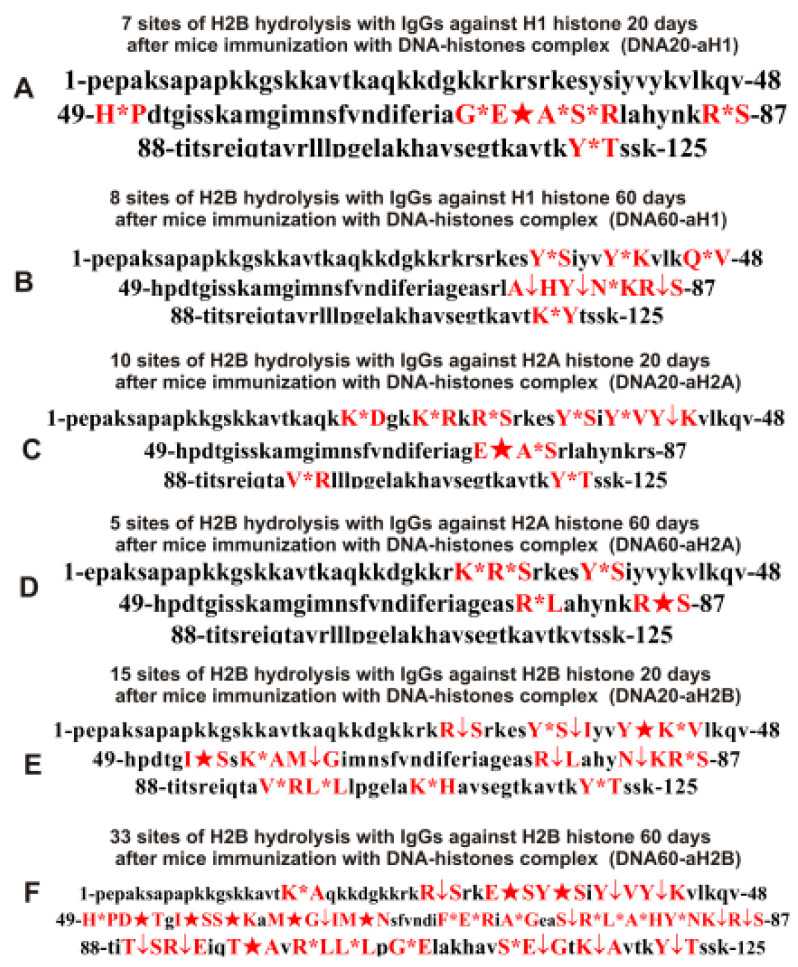
Sites of H2B hydrolysis by IgGs against three individual H1-H2B histones corresponding to 20 and 60 days after mouse immunization with DNA-histone complex: DNA20-aH1 (**A**), DNA60-aH1 (**B**), DNA20-aH2A (**C**), DNA60-aH2A (**D**), DNA20-aH2B (**E**), and DNA60-aH2B (**F**). Major sites of H2A cleavage are shown by stars (★), moderate sites by arrows (↓), and minor sites of cleavage by small stars (*).

**Figure 11:**
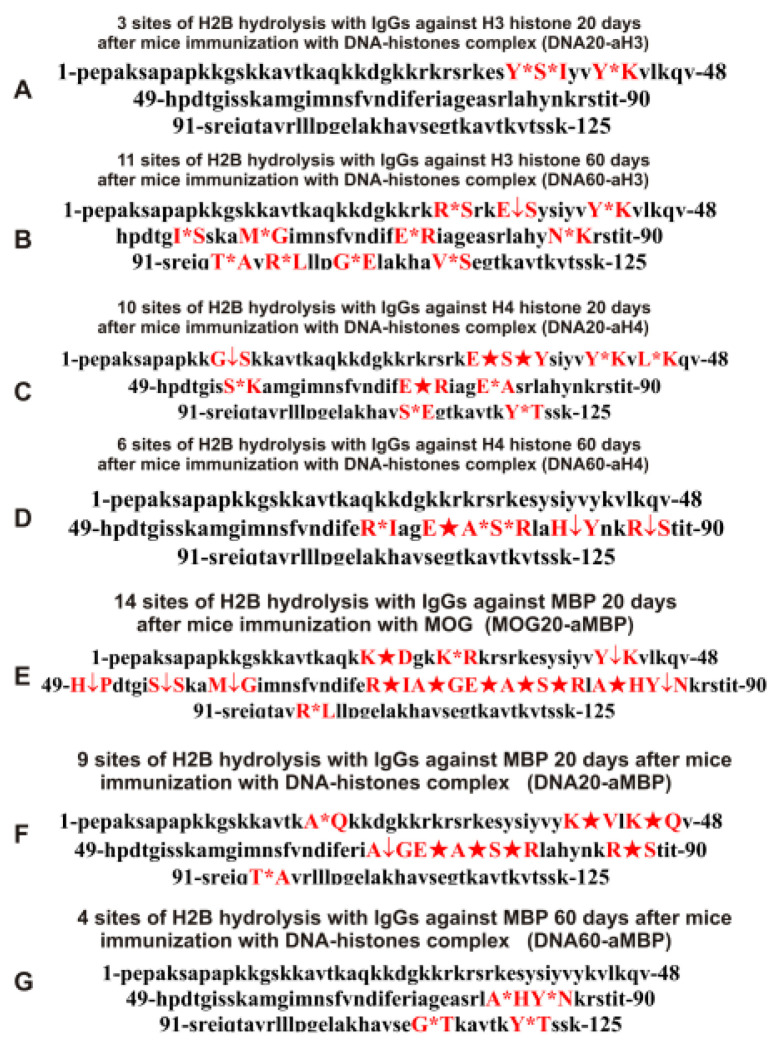
Sites of H2B hydrolysis by IgGs against H3 and H4 histones corresponding to 20 and 60 days after mouse immunization with DNA-histone complex: DNA20-aH3 (**A**), DNA60-aH3 (**B**), DNA20-aH4 (**C**), DNA60-aH4 (**D**), and anti-MBP IgGs 20 days after mouse immunization with MOG-MOG20-aMBP (**E**), as well as 20 and 60 days after mouse immunization with DNA-histone complex DNA20-aMBP (**F**) and DNA60-aMBP (**G**). Major sites of H2A cleavage are shown by stars (★), moderate sites by arrows (↓), and minor sites of cleavage by small stars (*).

The data obtained indicated an extreme diversity of abzymes against the five histones and MBP and a change in their substrate specificity in relation to different parts of the H2B protein sequence depending on the stage of EAE development. Overall, the sites of H2B histone cleavage by IgGs against the five histones H1-H4 and MBP corresponding to the beginning of the experiment (3-month-age mice), 60 days of spontaneous development of EAE, 20 days after mouse treatment with MOG, as well as 20 and 60 days after immunization with DNA-histone complex were substantially or very different and were predominantly located in specific amino acid (AA) clusters of different lengths (Figure 7, Figure 8, Figure 9, Figure 10 and Figure 11).

### 2.6. Tables Corresponding Sites of H2B Histone Hydrolysis

All IgG preparations split H2B histone in different numbers and types of sites. For easier comparison of the difference in sites of H2B hydrolysis with various IgGs corresponding to the beginning of the experiment and after spontaneous development of EAE within 60 days, they sites are given in Table 2.

Antibodies against H1 histone corresponding to 3-month-old mice (zero time, Con-aH1-0d) hydrolyzed this histone at 4 sites and at 28 sites after 60 days of spontaneous EAE development (Spont-aH1-60d); for these preparations, only 1 site was the same (Table 2).

During the spontaneous development of EAE, the number of histone H2B hydrolysis sites by antibodies against H2A decreased from 9 (Con-aH2A-0d) to 4 (Spont-aH2A-60d) and only 2 sites were the same (Table 2).

The number of sites of H2B hydrolysis by IgGs against H2B histone (Con-aH2B-0d) at zero time was only 6, but this number increased to 10 sites after 60 days of spontaneous development of EAE (Spont-aH2B-60d); again, only 2 sites were the same (Table 2).

The number of H2B hydrolysis sites for Spont-aH3-60d increased in comparison with Con-aH3-0d from 6 to 15. There were three identical sites, which differed in the efficiency of hydrolysis (Table 2).

The number of H2B hydrolysis sites with IgGs against H4 histone (Con-aH4-0d) increased from 6 to 7 after 60 days of spontaneous development of EAE. However, all sites of hydrolysis were different, except for one (Table 2).

When analyzing these data, it should be taken into account that a change in the differentiation profile of stem cells leads to the production of very different B lymphocytes producing abzymes already at the level of the cerebrospinal fluid of the bone marrow [38,39,40]. IgG-abzymes isolated from the cerebrospinal fluid of the bone marrow of patients with MS demonstrated 30–60 times higher activity in the hydrolysis of DNA, MBP, and oligosaccharides compared with Abs from the blood of the same patients [38,39,40]. In addition, at different stages of spontaneous and antigen-induced development of SLE and EAE, changes in the differentiation profile of bone marrow stem cells occurred at least three times [11,12,13,14,15,16,17].

Thus, during the spontaneous development of EAE, there may be expansion or constriction of the repertoire of lymphocytes producing abzymes with completely different properties compared to the beginning of EAE. Interestingly, the pools of antibodies against the five H1-H4 histones contained IgGs hydrolyzing H2B at the same sites as Abs corresponding to zero time but with more or less efficiency in the splitting this histone or hydrolyzing H2B at completely different sites.

The treatment of mice with MOG and DNA-histone complex led to a change in the profile of stem cell differentiation compared to the spontaneous development of EAE and, therefore, the appearance of IgGs against the five H1-H4 histones with completely different catalytic properties in the hydrolysis of H2B histone. As mentioned above, treatment of C57BL/6 mice with MOG led to a sharp acceleration in the development of EAE up to 20 days, corresponding to the acute phase, with subsequent remission of the pathology at >25 days [11,12,13,14]. Table 3 demonstrates data on the sites of hydrolysis of H2B histone by antibodies against the five different histones and MBP corresponding to 20 days after mouse immunization with MOG.

At time zero, the number of sites of hydrolysis of H2B by IgGs against H1 histone was 4 (Con-aH1-0d; Table 2), which was the same at 20 days after mouse immunization with MOG-26 (MOG20-aH1; Table 3). Interestingly, only one site was common for these IgGs. After 60 days of spontaneous development of EAE, the number of sites of H2B hydrolysis with IgGs against H1 was 28 (Spont-aH1-60d; Table 2) and only 9 of them were the same as the hydrolysis sites of H2B with MOG20-aH1 (Table 3).

The number of sites of H2B hydrolysis by anti-H2A antibodies at zero time (Con-aH2A-0d; Table 2) was 9, it increased after mouse treatment with MOG (MOG20-aH2A; Table 3) to 26, and 4 sites out of 9 coincided with those for MOG20-aH2A. Of the 4 sites of H2B hydrolysis by anti-H2A antibodies corresponding to 60 days of spontaneous EAE development (Spont-aH2A-60d; Table 2), 1 site coincided with that for MOG20-aH2A (Table 3).

The number of sites of H2B hydrolysis by IgGs against H2B histone at time zero was 6 (Con-aH2B-0d; Table 1), it increased to 7 after mouse treatment with MOG (MOG20-aH2B; Table 3), and only 2 sites were the same (Table 3). The spontaneous development of EAE led to an increase in the number of H2B hydrolysis sites by anti-H2B IgGs (Spont-aH2B-60d; Table 1) to 10. Of the 10 sites corresponding to the spontaneous development of EAE within 60 days (Spont-aH2B-60d; Table 2), only 1 site coincided with that for MOG20-aH2B antibodies (Table 3).

Immunization of mice with MOG (6 sites, MOG20-aH3; Table 3) did not lead to a change in the number of H2B hydrolysis sites with IgGs against H3 histone (6 sites, Con-aH3-0d; Table 2), but only one site was common for these IgGs. Of the 15 sites corresponding to hydrolysis of H2B by IgGs against H3 histone after 60 days of spontaneous EAE development (Spont-aH2B-60d; Table 2), 1 site coincided with that for MOG20-aH3 (Table 3).

With the spontaneous development of EAE, the number of H2B hydrolysis sites for anti-H4 IgGs increased from 6 to 7 (Table 2), while it increases to 11 after immunization of mice with MOG (MOG20-aH4) (Table 3), and there were no identical hydrolysis sites for this IgG and Con-aH4-0d (Table 2). However, MOG20-aH4 (Table 3) and Spont-aH4-60d (Table 2) had 2 identical sites.

Thus, immunization of mice with MOG significantly changed the set of lymphocytes producing IgGs against the five H1-H4 histones, which were able to hydrolyze H2B at different sites.

As previously shown, all five histones shared a high level of protein sequence homology with each other and with myelin basic protein. This is most likely the main reason for the cross-hydrolysis of H2B histone by antibodies against the five histones and MBP (Figure 1).

IgG antibodies against MBP effectively hydrolyzed H2B histone. The sites of H2B histone hydrolysis by different IgG preparations against MBP are given in Table 3.

Antibodies of 3-month-old mice against MBP (Con-aMBP) hydrolyzed H2A histone at 11 sites (Table 3). Twenty days after immunization of mice with MOG (MOG20-aMBP), the number of hydrolysis sites was 14. Two sites of H2B hydrolysis with these IgGs were the same, but they differed in hydrolysis efficiency (Table 3).

Overall, immunization of mice with MOG and DNA-histone complex led to a strong change in sites of H2B hydrolysis by various IgGs and many of them did not coincide.

Changes in the sites of H2B hydrolysis by IgGs against the five histones corresponding to 20 and 60 days after mouse immunization with DNA-histone complex are shown in Table 4.

If at zero time IgGs against H1 hydrolyzed H2B histone at 4 sites (Con-aH1-0d) and hydrolysis occurred at 28 sites after 60 days of spontaneous development of EAE (Spont-aH1-60d) (Table 2), then the number of hydrolysis sites decreased to 7 and 8 sites for 20 (DNA20-aH1) and 60 (DNA60-aH1) days after mouse treatment with DNA–histones complex, respectively (Table 4). Thus, the spontaneous development of EAE led to a significant expansion of the repertoire of lymphocytes producing IgGs against H1 histone hydrolyzing H2B histone, while immunization of mice with DNA-histone complex resulted in a moderate increase in the number of H2B hydrolysis sites (Table 2 and Table 4). Immunization of mice with DNA-histone complex led to a significant change in the sites of H2B hydrolysis by antibodies against H1 histone. Interestingly, none of the 4 sites of H2B hydrolysis by Con-aH1-0d antibodies coincided with those for DNA20-aH1 (Table 2 and Table 4). Only 4 H2B hydrolysis sites by DNA60-aH1 were found among the 26 sites of histone hydrolysis by the Spont-aH1-60d preparation (Table 2 and Table 4). From 20 to 60 days after mouse immunization with DNA-histone complex, significant changes also occurred in the production of anti-H1 IgGs capable of hydrolyzing H2B. Only 1 hydrolysis site was common for DNA20-aH1 and DNA60-aH1 (Table 4). Both spontaneous development of EAE (28 sites, Table 2) and immunization of mice with MOG (26 sites, Table 3) led to a significant increase in the number of H2B hydrolysis sites with IgGs against H1 histone. It is interesting that there were no common sites for MOG20-aH1 (20 days after immunization of mice with MOG) with DNA20-aH1, but 2 of the same sites were observed with the DNA60-aH1 preparation corresponding to 60 days after mouse treatment with DNA-histone complex (Table 3 and Table 4).

At time zero, IgGs against H2A (Con-aH2A0d) hydrolyzed the H2B histone at 8 sites (Table 2). The number of H2B hydrolysis sites with anti-H2A IgGs at 20 days after immunization of mice with DNA-histone complex (DNA20-aH2A) increased to 10, but then decreased to 5 for 60 days after mouse treatment (DNA60-aH2A; Table 4). For DNA20-aH2A and DNA60-aH2A, one common hydrolysis site was found (Table 4). In the case of the anti-H2A preparation corresponding to zero time (Con-aH2A-0d), there were no coinciding hydrolysis sites with those for DNA20-aH2A and only one site was identical to that for DNA60-aH2A. For the Spont-aH2A-60d preparation corresponding to the spontaneous development of EAE (Table 2), there was one coinciding site of H2B hydrolysis with the DNA20-aH2A and DNA60-aH2A preparations (Table 4). Of the 26 sites of H2B hydrolysis by MOG20-aH2A antibodies (Table 3), only 4 and 3 sites coincided with those for the DNA20-aH2A and DNA60-aH2A preparations, respectively (Table 4).

During the spontaneous development of EAE from time zero (Con-aH2B-0d) to 60 days, the number of sites of H2B hydrolysis by IgGs against H2B histone (Spont-aH2B-60d) increased from 6 to 10 (Table 2). Interestingly, the treatment of mice with DNA-histone complex led to an increase in the number of H2B hydrolysis sites to 15 and 33 for 20 (DNA20-aH2B) and 60 (DNA60-aH2B) days after mouse immunization with DNA-histone complex (Table 4). Only 9 of the same H2B hydrolysis sites with DNA20-aH2B and DNA60-aH2B were found. Among the 15 H2B hydrolysis sites for Con-aH2B-0d, 2 coincided with those for DNA20-aH2B and 4 for DNA60-aH2B. All H2B hydrolysis sites for Spont-aH2B-60d (Table 2) and DNA20-aH2B (Table 4) were different. However, among 33 H2B hydrolysis sites for DNA60-aH2B, 7 coincided with those for Spont-aH2B-60d. Of the 7 sites of H2B hydrolysis by IgGs after mouse immunization with MOG (MOG20-aH2B; Table 3), 2 and 4 sites coincided with those corresponding to DNA20-aH2B and DNA60-aH2B, respectively (Table 4). Thus, the treatment of mice with DNA-histone complex strongly affected the changes in the differentiation profile of stem cells, leading to the production of lymphocytes producing IgGs against H2B histone capable of hydrolyzing H2B at different sites.

With the spontaneous development of EAE, the appearance of lymphocytes synthesizing antibodies against different histones was entirely different. If the number of antibodies against H1, H2B, and H3 hydrolyzing H2B histone increased during spontaneous development of EAE, in the case of other IgGs, it decreased or remained nearly the same (Table 2). Interestingly, compared to zero time (Con-aH3-0d; Table 2) with 6 sites of H2B hydrolysis by IgGs against H3 histone, this number decreased to 3 sites for 20 days after treatment of mice with DNA-histone complex (DNA20-aH3) (Table 4). However, the effect of immunization with DNA-histone complex turned out to be multistage, and by 60 days after immunization, the number of hydrolysis sites was increased to 11 (Table 4). Nevertheless, even 20 days after immunization, there were noticeable changes in hydrolysis sites; all three sites in the case of DNA20-aH3 were new compared to those for Con-aH3-0d (Table 2 and Table 4). Of the 15 and 11 sites of H2B hydrolysis by DNA60-aH3 and Spont-aH3-60d, only 2 sites coincided (Table 2 and Table 4). Among the hydrolysis sites of the corresponding IgG after mouse immunization with MOG (MOG20-aH3, Table 3), only 1 and 2 sites coincided with those for DNA20-aH3 and DNA60-aH3, respectively (Table 4). Thus, in the case of antibodies against H3 hydrolyzing H2B histone, different patterns of changes of H2B hydrolysis were observed after spontaneous and MOG- and DNA-histone complex-induced development of EAE compared to IgGs against the three other histones (H1, H2A, and H2B) described above.

The number of sites of hydrolysis of H2B by IgGs against H4 histone practically did not change during 60 days of spontaneous development compared with zero time; 6 (Con-aH4-0d) and 7 (Spont-aH4-60d) sites were found, respectively (Table 2). Interestingly, the number of sites of H2B hydrolysis by antibodies against H4 histone changed to 10 and 6 sites 20 (DNA20-aH4) and 60 (DNA60-aH4) days after immunization with DNA-histone complex (Table 4). There were no common hydrolysis sites for DNA20-aH4 with those for Con-aH4-0d, but there were 2 of the same sites with Spont-aH4-60d (Table 2 and Table 4). Only one site of hydrolysis of H2B by IgGs against H4 histone was found at 20 days (DNA20-aH4) and 60 (DNA60-aH4) days after immunization with DNA-histone complex (Table 4). In the case of the MOG20-aH4 preparation corresponding to immunization of mice with MOG (Table 3), only one site coincided with that for DNA20-aH4 and DNA60-aH4 antibodies (Table 4).

Thus, compared to spontaneous development of EAE, immunization of mice with MOG and DNA-histone complex induced very different changes in the repertoire of lymphocytes producing antibodies against H1, H2A, H2B, H3, and H4 histones, which were capable of hydrolyzing H2B. The patterns of H2B hydrolysis with all IgG preparations differed in the number, efficiency, and type of cleavage sites (Table 2, Table 3 and Table 4).

## 3. Discussion

The polyspecificity or polyreactivity of the formation of only partially specific complexes characterizing different antibodies is widespread [62,63,64,65]. The affinity of Abs for partially non-specific foreign antigens is commonly significantly lower than that for completely specific cognate ones, and such Abs can be traditionally removed from affinity sorbents by 0.1–0.15 M NaCl [19,20,21,22,23]. Therefore, we eluted non-specifically bound Abs from affinity columns using 0.2 M NaCl. In addition, IgGs against the five histones and MBP were passed through alternative affinity sorbents. Then, the fraction containing IgGs against the five histones was used to isolate Abs against each of the five individual histones. Finally, IgG fractions were obtained against all five histones and MBP containing no alternative IgGs.

It was previously proven that polyclonal preparations of IgGs from the EAE-prone C57BL/6 mice used in this study did not contain any canonical proteases [47]. As previously shown, the pools of monoclonal IgGs of patients with SLE contained Abzs with serine-like, metal-dependent, and thiol-like proteolytic activities, which, in contrast to canonical proteases, hydrolyzed proteins not on all possible sites of cleavage but only at specific sequences for which they had increased affinity [67,68,69,70,71,72]. The comparison of H2B cleavage sites with abzymes against the five individual histones (H1-H4) and MBP well endorsed this conclusion (Figure 7, Figure 8, Figure 9, Figure 10 and Figure 11, Table 2, Table 3 and Table 4). Trypsin splits different proteins after arginine (R) and lysine (K) residues. The H2B sequence contains 20 sites for potential cleavage of H2B by trypsin after K and 5 sites after R. Abzymes hydrolyze H2B not at all possible sites but at a limited number of sites after K and R, which does not correspond to the entire protein sequence but is located in specific clusters of the H2B protein sequence. In addition, the specific sites of H2B cleavage after K and R for all IgG preparations against MBP and the five individual histones were very different and corresponded to different specific clusters (Figure 2, Figure 3, Figure 4, Figure 5, Figure 6, Figure 7, Figure 8, Figure 9, Figure 10 and Figure 11 and Table 2, Table 3 and Table 4). This indicated that the pools of IgGs against MBP and the five individual histones (H2B, H1, H2A, H3, and H4) contained small fractions of histone sequence-specific serine-like abzymes.

Chymotrypsin hydrolyzes proteins after F, Y, and W aromatic AAs. The H2B protein sequence contains two residues of F and five of Y. Only one IgG preparation (Con-aH1-60d, Table 2) cleaved H2B after F (minor site). This could, to some extent, indicate the absence of IgGs with active centers similar to classical chymotrypsin in the preparations against MBP and the five histones. However, several IgGs hydrolyzed H2B after Y (Figure 7, Figure 8, Figure 9, Figure 10 and Figure 11, Table 2, Table 3 and Table 4). Therefore, it is not entirely clear whether the active sites of these abzymes are similar to canonical chymotrypsin or whether they have other active centers more specific to Y but not to F. However, it is still impossible to exclude the possibility that some small fraction of polyclonal abzymes had active centers more or less similar to chymotrypsin.

The obtained data on H2B cleavage sites indicated that, in addition to IgGs with serine-like active centers that could mimic those of trypsin and chymotrypsin, there were IgG-abzymes with other alternative active centers in the pool of IgGs against the five histones and MBP. In contrast to canonical proteases, the main hydrolysis sites were substantially grouped in specific clusters of the H2B histone sequence. They mainly occurred after neutral amino acids Q, T, G, A, L, D, I, M, N, T, E, S, and V (Figure 7, Figure 8, Figure 9, Figure 10 and Figure 11, Table 2, Table 3 and Table 4). The specific sites of H2B cleavage by IgG preparations against the five individual histones and MBP were not located along the entire H2B histone length but were mainly grouped into particular amino acid clusters. Depending on the IgG preparation, they may be located in very different or overlapping clusters of the H2B protein sequence (Figure 7, Figure 8, Figure 9, Figure 10 and Figure 11, Table 2, Table 3 and Table 4).

The strong proof that the abzymes against MBP and the five individual histones did not contain even perceptible impurities of IgGs against alternative histones arises from the very different number of specific sites of H2B hydrolysis (from 3 to 33) by IgG-abzymes against each of the five individual histones and MBP (Figure 7, Figure 8, Figure 9, Figure 10 and Figure 11, Table 2, Table 3 and Table 4). Interestingly, the same major hydrolysis sites were found for some abzyme preparations as well as very specific individual ones, but there was not even one common hydrolysis site for all 29 IgG preparations (Table 2, Table 3 and Table 4). All cleavage sites differed not only in their location in the H2B sequence but the same hydrolysis sites for several different IgGs varied significantly in their relative efficiency of H2B splitting, with major, medium, or minor sites (Figure 7, Figure 8, Figure 9, Figure 10 and Figure 11, Table 2, Table 3 and Table 4).

The analysis of which hydrolysis sites are most often considered major in the case of all preparations used is of particular interest. Different major sites were common among H2B hydrolysis sites in the case of different IgG preparations (number of preparations): E35-S36, I54-S55, and E76-A77 (4), S38-I39, Y42-K43, and R86-S87 (3), Y37-S38, Q47-V48, M59-G60, M62-N63, E71-R72, E113-G114, T119-K120, K20-A2, and R31-S32 (2). Interestingly, only one original major H2B hydrolysis site was found in the case of 17 IgG preparations (Table 2, Table 3 and Table 4). Therefore, it is interesting to understand why at different stages of spontaneous EAE development and after immunization of mice with MOG and DNA-histone complex, the formation of IgGs hydrolyzing H2B histone at many different sites is possible. For this purpose, it is helpful to take into account some literature data.

First, MS is at least a two-phase autoimmune disease { XE “pathology” } [73]. The cascade of many reactions in the first inflammatory { XE “inflammatory” } phase is very sophisticated, involving many cytokines { XE “cytokines” }, chemokines, enzymes { XE “enzymes” }{ XE “chemokines” }, proteins, and other compounds inducing macrophages { XE “macrophages” } and other cells producing NO { XE “NO” } radicals and osteopathin [73]. The coordinated constituent action of B and T cell { XE “B-cell” } s { XE “B-cells” }, complement { XE “complement” } systems { XE “complement system” }, mediators of inflammation { XE “inflammation” }{ XE “mediators” }, and auto-antibodies leads to the formation of demyelination { XE “demyelination” } nidi and infringement of axon { XE “axon” } conductivity { XE “conductivity” }. The neurodegenerative { XE “neurodegenerative” } stage of MS that appears later is directly associated with the patient’s neural tissue { XE “neural tissue” }{ XE “tissue” } destruction { XE “destruction” } [73]. Therefore, during the analysis of immunological, biochemical, { XE “immunological” } and clinical indices of MS, every current phase of the disease must be considered, including changes in immunoregulation { XE “immunoregulation” }, exhaustion of different compensatory and adaptive mechanisms, and systemic metabolic { XE “metabolic” } changes [73]. It should be assumed that spontaneous and antigen-induced development of EAE in mice can also proceed in several stages, and autoantigens, including histones and MBP, can form many different complexes with a variety of proteins, nucleic acids, lipids, polysaccharides, cells, etc. at different stages.

When analyzing the diversity in the number and type of sites of H2B hydrolysis by Abs against the five individual histones and MBP, it is important to take into account that spontaneous and antigen-induced development of autoimmune reactions associated with changes in the differentiation profile of bone marrow stem cells also occur in several stages. At the beginning and acute phase, there are the first and second steps, and with the transition to a deep pathology, the next step in changes to the differentiation profile is observed [11,12,13,14,15,16,17]. Treatment of mice with MOG and DNA-histone complex strongly alters the pattern of HSC differentiation compared to its transformation during the spontaneous development of EAE [11,12,13,14,15,16,17].

It is known that the formation of natural abzymes occurs against specific structures of various molecules that imitate, at least to some extent, the transition states of chemical reactions [18,19,20,21,22,23]. In principle, abzymes can be formed in the case of any of the antigenic determinants or sequences of proteins that mimic the transition state of a hydrolysis reaction of peptide bonds. In addition, a variety of compounds can affect the immunogenicity of individual or associate histones, improving or impairing their ability to stimulate antibody and abzyme production.

Importantly, all histones have several antigenic determinants (AGDs) [74,75,76,77]. H2B histone has at least seven antigenic regions [78], while MBP has four AGDs [79,80]. At different stages of EAE development, each of the histones and their complexes with each other as well as with DNA or other molecules or cells can represent different fragments of protein sequences that mimic the transition states of the hydrolysis of peptide bonds for the formation of abzymes. In addition, all these molecules and their associates at different stages of EAE development can form various complexes with some proteins, oligosaccharides, lipids, etc., which can lead to the screening of some protein determinants and the formation of new sequences imitating transition states. This may be one of possible reasons for the formation of very different abzymes hydrolyzing H2B at different sites during various stages of EAE development.

It is important that all antigenic determinants of histones have a level of high homology between themselves and with those of MBP [44,45,46]. Therefore, it was estimated that general homology would be shared between the protein sequence of H2B with that of the four other histones and MBP. According to five alignment of the sequences, different fragments of H2B demonstrated the following comparable complete identity of amino acids between H2B and the four histones H1-H4 and similarity with non-identical amino acids together with highly similar physicochemical properties (%): H1: 22.2–40.0 (45.5–72.4), H2A: 21.6–43.8 (55.7–81.2), H3: 22.1–42.9 (51.9–69.2), and H4: 19.6–50 (51.7–80.0). Since IgGs against MBP efficiently hydrolyze H2B histone, we estimated the homology of the MBP sequence with that of H2B. H2B histone has 23.1–75.0% identity of amino acids with MBP and 46.0–87.5% similarity. The indicators of amino acid identity and similarity of the sequences of H2B with all four histones and MBP were very similar. Like histones, MBP contains many positively charged amino acid residues and binds efficiently to DNA [81]. Thus, this may be the second reason why protein sequences of histones and MBP with altered structures in various types of complexes can mimic similar transition states of hydrolytic reactions and stimulate the formation of abzymes with catalytic cross-reactivity.

At all stages of EAE development, apoptosis of various cells constantly occurs and chromatin DNA-histone complexes enter the blood [3]. This leads to the production of Abs against DNA itself, individual histones, or their complexes. However, there may be quite different variants of such complexes between the five histones. DNA in complex with histones can sterically shield some epitopes and, at the same time, increase the immunogenicity of other sequences of histones available for the stimulation of abzyme formation. In addition, the five histones within such complexes can form new antigenic determinants at the junction of two or even more histones. In this case, recognition sites of abzymes may be chimerically capable of binding and hydrolyzing several histones.

It has recently been shown that antigenic determinants are formed at the junction of protein sequences of histones and DNA, the formation of antibodies against which leads to the production of abzymes that hydrolyze both DNA and histones [72]. This can lead to the formation of a massive number of very different abzymes against individual histones and their complexes with each other and other blood components. Efficient hydrolysis of histone H4 by EAE-prone mice IgGs against the five histones H1-H4 has recently been shown; antibodies against all five histones and MBP effectively hydrolyzed H4 histone [47]. In addition to the above pathways for the formation of antibodies demonstrating catalytic cross-reactivity against various histones and MBP, another reason for this phenomenon may be the high level of protein sequence homology of all five histones and MBP [45,46]. Thus, the formation of polyreactivity of complexation and catalytic cross-reactivity of abzymes against histones and MBP can occur in several parallel ways.

## 4. Materials and Methods

### 4.1. Materials and Chemicals

All compounds used, H1 (M2501S), H2A (M2502S), H2B (M2508S), H4 (M2504S), H3 (M2503S), and an equimolar mixture of the histones (H9250) were from Sigma (St. Louis, MO, USA). Protein G-Sepharose (17061801) and Superdex 200 HR 10/30 columns (17061801) were from GE Healthcare (New York, NY, USA). MBP was obtained from the Center of Molecular Diagnostics and Therapy (DBRC-HMBP; Moscow, Russia). Affinity sorbents containing immobilized MOG, the mixture of the five histones, or the five individual histones were obtained according to the standard manufacturer’s protocol using BrCN-activated Sepharose from GE Healthcare (17098101), the mixture of the five histones, the five individual histones, or MBP. Mouse oligopeptide MOG_35-55_ was bought from EZBiolab (Heidelberg, Germany). All preparations used were free from any possible contaminants.

### 4.2. Experimental Animals

3-month-old inbred C57BL/6 mice were used by us recently to analyze the possible mechanisms of spontaneous and antigen-induced development of EAE [11,12,13,14,47]. They were raised using standard conditions free of pathogens in a special mouse vivarium at the Institute of Cytology and Genetics (ICG). All experiments with C57BL/6 mice were implemented under the ICG protocols of the Bioethical Committee (number of the document—134A of 7 September 2010), satisfying the humane principles for experiments with animals of European Communities Council Directive: 86/609/CEE. This Committee of the Institute supported this study. The relative weights, titers of Abs against histones and MBP, concentration of urine proteins in mg/mL (proteinuria), and some other indexes characterizing progress in EAE development were analyzed and previously described [11,12,13,14,47].

### 4.3. Antibody Purification

Electrophoretically homogeneous preparations of polyclonal IgGs from the blood plasma of C57BL/6 mice were first isolated by plasma protein affinity chromatography on Protein G-Sepharose. The IgG preparations were additionally purified by FPLC (fast protein liquid chromatography–gel filtration) on the Superdex-200 HR 10/30 column [11,12,13,14,47]. After gel filtration for additional purification of IgGs, the central parts of the Abs peaks were passed through filters (pore size 0.1 µm) as in ref. [11,12,13,14,47].

Removal of all antibodies against the five histones (H1-H4) from the total IgG preparations was fulfilled using histone5His-Sepharose (5 mL) containing the immobilized five H1-H4 histones. The column was equilibrated with buffer A (20 mM Tris-HCl, pH 7.5). After antibody loading, the column was washed with buffer A to zero optical density (A_280_). Non-specifically adsorbed antibodies possessing a low affinity for the five histones were eluted using buffer A supplemented with NaCl (0.2 M). Then, IgGs with high affinity for the histones were desorbed specifically using an acidic buffer (0.1 M glycine-HCl, pH 2.6). The IgGs eluted from histone5His-Sepharose at loading and washing of the column with buffer A (5 mL) were unified and used to isolate IgGs against MBP by chromatography on the 5 mL MBP-Sepharose column equilibrated with buffer A. After washing MBP-Sepharose with buffer A to zero optical density (A_280_), adsorbed IgGs with low affinity for MBP were eluted first using buffer A and then with this buffer containing NaCl (0.2 M). Then, anti-MBP IgGs were eluted from the sorbent by acidic Tris-Gly buffer (pH = 2.6), similar to the histone5His-Sepharose. Further, this fraction of antibodies was named and used as anti-MBP antibodies. Such IgGs were obtained in the case of mice corresponding to different stages of development of EAE before (Con-aMBP-0d, Spont-aMBP-60d) and after immunization of mice with MOG (MOG20-aMBP) and DNA-histone complex (DNA20-aMBP and DNA60-aMBP) (Table 1).

For additional purifications of IgGs against the five histones from possible hypothetical small impurities of Abs against MBP, the fractions eluted from histone5His-Sepharose were re-chromatographed on MBP-Sepharose. IgGs eluted after loading on MBP-Sepharose were named anti-5-histones IgGs. IgGs against the five histones were obtained from plasma of the blood of mice corresponding to different stages of development of EAE before (Con-aH1-H4-0d, Spont-aH1-H4-60d) and after immunization of mice with MOG (MOG20-aH1-H4-20) and DNA-histone complex (DNA20-aH1-H4 and DNA60-aH1-H4) (Table 1).

### 4.4. Antibody Purification against Individual Five Histones

All five preparations against the five histones, corresponding to different stages of EAE development, were further used to obtain IgGs against each of the five individual histones. All of them were applied sequentially first on H2B-Sepharose containing immobilized H2B histone. The fractions eluted at loading were then applied sequentially to the following four sorbents: H1-Sepharose, H2A-Sepharose, H3-Sepharose, and H4-Sepharose. All affinity chromatography was performed as in the case of histone5His-Sepharose and MBP-Sepharose. IgGs against H2B and other histones were specifically desorbed from each of five affinity sorbents with buffer, pH 2.6. These IgG fractions were designated respectively as anti-H2B, anti-H1, anti-H2A, anti-H3, and anti-H4 IgGs, with the denotation to which stage and which antigen they corresponded: Con (beginning of the experiment); Spont (spontaneous of EAE development); MOG (mice treated with MOG); DNA (mice treated with DNA-histone complex) (Table 1).

### 4.5. Proteolytic Activity Assay

The protease activity of all IgG-abzymes was analyzed by SDS-PAGE using the reaction mixtures (10–18 μL) containing Tris-HCl buffer (20 mM; pH 7.5), 0.9–1.0 mg/mL MBP, the mixture of the five histones, or H2A histone, and 0.012 mg/mL IgGs against MBP (0.07–0.1 mg/mL) or the five histones, as described in ref. [47]. All mixtures of histones and IgGs were incubated for 3–14 h at 37 °C. Then, all reactions were stopped by adding SDS to the 0.1% final concentration. The efficiencies of H2B and MBP hydrolysis were analyzed by SDS-PAGE in 20% gel. All gels were colored using silver or Coomassie Blue. The relative protease activities of IgGs were evaluated from the decrease in the relative intensity of protein bands corresponding to initial non-hydrolyzed H2B or MBP corresponding to these proteins incubated without Abs. A more detailed analysis of the hydrolysis of H2B by all obtained Abs was carried out using MALDI-TOF spectrometry.

### 4.6. MALDI-TOF Analysis of Histone Hydrolysis

H2B histone was hydrolyzed for 0–20 h using all preparations of anti-MBP and against the five individual histone IgGs using the conditions described above. The H2A histone hydrolysis products were ascertained by the 337 nm nitrogen laser VSL-337 ND with a 3 ns pulse duration using the Reflex III system (Bruker, Frankfurt, Germany). Small aliquots of the reaction mixtures (1–2 µL) were used for analysis by MALDI mass spectrometry after incubation of the reaction mixtures at different times. Sinapinic acid was used as the matrix. To 1.7 µL of the matrix and 1.7 µL of 0.2% trifluoroacetic acid, 1.7 µL of the solution containing histone H2B was added, and 1–1.7 µL of the obtained mixture was applied to the iron MALDI plates. For the analysis, the plates were air-dried. All MALDI spectra were calibrated using standards II and I calibrant mixtures of special oligopeptides and proteins (Bremen, Germany, Bruker Daltonic) in the external and/or internal calibration mode. The analysis of molecular weights and specific sites of H2B hydrolysis by various IgGs was performed using Protein Calculator v3.3 (Scripps Research Institute; San Diego, USA).

### 4.7. Analysis of Sequence Homology

The analysis of protein sequence homology between histones and MBP was performed using *lalign* (http://www.ch.embnet.org/software/LALIGN_form.html, accessed on 10 September 1998).

### 4.8. Statistical Analysis

The results corresponded to the average values (mean ± standard deviation) from 8–10 independent spectra for each preparation of IgGs against the five individual histones and MBP.

## 5. Conclusions

Here, we have demonstrated for the first time that IgG-abzymes from EAE-prone C57BL/6 mice against five individual histones and myelin basic protein can form complexes with H2B histone, thereby showing polyreactivity in complexation. In addition, all IgG preparations possessed unusual enzymatic cross-reactivity in the hydrolysis of histone H2B. IgGs against the five individual histones and MBP at the beginning of the experiment (3-month-old mice) and after spontaneous development of EAE during 60 days differed in the relative number of hydrolysis sites and their type. After mouse immunization with MOG or DNA-histone complex, the sites of H2B hydrolysis differed from each other as well as from those corresponding to the beginning of the experiment and spontaneous development of EAE. Overall, the number of H2B hydrolysis sites depended on the IgG preparation and varied from 3 to 33. The treatment of mice with MOG and DNA-histone complex accelerated EAE evolution and led to a change in the relative activity of IgGs in H2B hydrolysis. Possible reasons for the catalytic cross-reactivity of antibodies and such strong differences in the number and type of histone H2B cleavage sites were analyzed.

## Figures and Tables

**Table 1:** IgGs against the five histones (total), MBP, and five individual histones corresponding to different stages of EAE development *.

**Zero time (control),** **beginning of experiments (3- month-old mice)**	**Different total IgGs**	Designation	IgGs against individual histones	Designation
	Zero time (control) beginning of the experiment, IgGs against 5 histones and MBP	Con-aH1-H4-0d **	**anti-H1 histone**	Con-aH1-0d
			**anti-H2A histone**	**Con-aH2A-0d**
			**anti-H2B histone**	**Con-aH2B-0d**
		Con-aMBP-0d	**anti-H3 histone**	**Con-aH3-0d**
			**anti-H4 histone**	**Con-aH4-0d**
**Spontaneous development of EAE during 60 days (without mouse immunization at 3 months of age)**	Spontaneous development of EAE during 60 days; IgGs against 5 histones and MBP	Spont-aH1-H4-60d	**anti-H1 histone**	Spont-aH1-60d
			**anti-H2A histone**	**Spont-aH2A-60d**
			**anti-H2B histone**	**Spont-aH2B-60d**
		Spont-aMBP-60d	**anti-H3 histone**	**Spont-aH3-60d**
			**anti-H4 histone**	**Spont-aH4-60d**
**IgGs corresponding to 20 days after mouse immunization with MOG**	IgGs against 5 histones and MBP corresponding to 20 days after mouse immunization with MOG	MOG20-aH1-H4-20	**anti-H1 histone**	MOG20-aH1
			**anti-H2A histone**	**MOG20-aH2A**
			**anti-H2B histone**	**MOG20-aH2B**
		MOG20-aMBP	**anti-H3 histone**	**MOG20-aH3**
			**anti-H4 histone**	**MOG20-aH4**
**IgGs corresponding to 20 days after mouse immunization with DNA-histone complex**	**IgGs against 5 histones and MBP; 20 days after mouse immunization with DNA-histone complex**	DNA20-aH1-H4	**anti-H1 histone**	DNA20-aH1
			**anti-H2A histone**	**DNA20-aH2A**
			**anti-H2B histone**	**DNA20-aH2B**
		**DNA20-aMBP**	**anti-H3 histone**	**DNA20-aH3**
			**anti-H4 histone**	**DNA20-aH4**
**IgGs corresponding to 60 days after mouse immunization with DNA-histone complex**	**IgGs against 5 histones and MBP; 60 days after mouse immunization with DNA-histone complex**	DNA60-aH1-H4	**anti-H1 histone**	DNA60-aH1
			**anti-H2A histone**	**DNA60-aH2A**
			**anti-H2B histone**	**DNA60-aH2B**
		**DNA60-aMBP**	**anti-H3 histone**	**DNA60-aH3**
			**anti-H4 histone**	**DNA60-aH4**

* All IgG preparations were used to analyze H2B histone hydrolysis. ** In terms of preparations, Con corresponds to control IgG preparations—zero time of the experiment (3-month-old mice); Spont-60—spontaneous development of EAE within 60 days after the start of the experiment; MOG20—20 days after mouse treatment with MOG; DNA20 and DNA60—20 and 60 days after mouse immunization with DNA-histone complex, respectively. In addition, the short designations of the preparations indicate the antigen of the antibody: aH1-aH4, anti-H1-anti-H4 five histones; aMBP, anti-MBP; aH1, anti-H1; aH2A, anti-H2A; aH2B, anti-H2B; aH3, anti-H3; aH4, anti-H4 against individual H1-H4 histones.

**Table 2:** Sites of H2B histone hydrolysis by IgGs against three individual histones at zero time and after spontaneous development of EAE during 60 days *.

Sites of H2B Hydrolysis at the Beginning of Experiment and after 60 Days of Spontaneous Development of EAE									
Con-aH1-0d	Spont-aH1-60d	Con-aH2A-0d	Spont-aH2A-60d	Con-aH2B-0d	Spont-aH2B-60d	Con- aH3-0d	Spont- aH3-60d	Con- aH4-0d	Spont- aH4-60d
4 Sites	28 Sites	9 Sites	6 Sites	6 Sites	10 Sites	6 Sites	15 Sites	6 Sites	7 Sites
**-**	**-**	**-**	**-**	**-**	**T19-K20**	**-**	**-**	**-**	**-**
**K20-A21**	**K20-A21**	**K20-A21**	**K20-A21**	**K20-A21**	**K20-A21**	**K20-A21**	**K20-A21**	**K20-A21**	**K20-A21**
**-**	**D25-G26**	**-**	**-**	**-**	**-**	**-**	**-**	**-**	**-**
**-**	**-**	**-**	**R33-K34**	**-**	**-**	**-**	**-**	**-**	**-**
**-**	**-**	**-**	**-**	**-**	**E35-S36**	**-**	**E35-S36**	**-**	**-**
**-**	**S36-Y37**	**-**		**-**	**S36-Y37**	**-**	**-**	**-**	**-**
**-**	**-**	**-**	**Y37-S38**	**-**	**-**	**-**	**-**	**-**	**-**
**S38-I39**	**-**	**S38-I39**	**-**	**S38-I39**	**-**	**-**	**-**	**-**	**-**
**-**	**-**	**-**	**-**	**-**	**-**	**-**	**-**	**-**	**-**
**-**	**-**	**-**	**Y40-V41**	**Y40-V41**	**-**	**-**	**-**	**-**	**Y40-V41**
**-**	**-**	**-**	**-**	**-**	**-**	**-**	**V41-Y42**	**-**	**-**
**Y42-K43**	**-**	**-**	**-**	**Y42-K43**	**-**	**-**	**-**	**-**	**Y42-K43**
**-**	**-**	**V44-L45**	**-**	**-**	**-**	**-**	**-**	**-**	**-**
**-**	**Q47-V48**	**-**	**-**	**-**	**-**	**-**	**Q47-V48**	**-**	**-**
**-**	**-**	**-**	**-**	**-**	**-**	**-**	**-**	**-**	**V48-H49**
**-**	**D51-T52**	**-**	**-**	**-**	**-**	**-**	**-**	**-**	**-**
**T52-G53**	**-**	**-**	**-**	**-**	**-**	**-**	**-**	**-**	**-**
**-**	**G53-I54**	**-**	**-**	**-**	**-**	**-**	**G53-I54**	**-**	**-**
**-**	**I54-S55**	**I54-S55**	**-**	**-**	**-**	**-**	**-**	**I54-S55**	**-**
**-**	**S55-S56**	**-**	**-**	**-**	**-**	**-**	**S55-S56**	**-**	**-**
**-**	**S56-K57**	**-**	**-**	**S56-K57**	**S56-K57**	**-**	**-**	**-**	**-**
**-**	**A58-M59**	**-**	**-**	**-**	**-**	**-**	**-**	**-**	**-**
**-**	**M59-G60**	**-**	**-**	**-**	**-**	**G60-I61**	**-**	**-**	**-**
**-**	**-**	**-**	**-**	**-**	**-**	**-**	**-**	**G60-I61**	**-**
**-**	**I61-M62**	**-**	**-**	**-**	**-**	**-**	**-**	**-**	**-**
**-**	**M62-N63**	**-**	**-**	**-**	**-**	**-**	**M62-N63**	**M62-N63**	**-**
**-**	**S64-F65**	**-**	**-**	**-**	**-**	**-**	**-**	**-**	**-**
**-**	**-**	**-**	**-**	**-**	**-**	**-**	**N67-D68**	**-**	**-**
**-**	**F70-E71**	**-**	**-**	**-**	**-**	**-**	**-**	**-**	**-**
**-**	**-**	**-**	**-**	**-**	**E71-R72**	**-**	**-**	**-**	**-**
**-**	**-**	**-**	**-**	**-**	**-**	**-**	**A74-G75**	**-**	**-**
**-**	**-**	**-**	**-**	**-**	**-**	**E76-A77**	**-**	**-**	**-**
**-**	**A77-S78**	**-**	**-**	**-**	**-**	**-**	**-**	**-**	**-**
**-**	**-**	**-**	**-**	**-**	**-**	**-**	**S78-R79**	**-**	**-**
**-**	**R79-L80**	**-**	**-**	**-**	**-**	**-**	**-**	**-**	**-**
**-**	**H82-Y83**	**-**	**-**	**-**	**-**	**-**	**-**	**-**	**-**
**-**	**Y83-N84**	**-**	**R79-L80**	**-**	**-**	**-**	**-**	**-**	**-**
**-**	**K85-R86**	**-**	**-**	**-**	**-**	**-**	**-**	**-**	**-**
**-**	**R86-S87**	**R86-S87**	**-**	**R86-S87**	**-**	**-**	**R86-S87**	**-**	**R86-S87**
**-**	**-**	**S87-T88**	**-**	**S87-T88**	**-**	**-**	**-**	**-**	**S87-T88**
**-**	**-**	**-**	**-**	**-**	**-**	**-**	**-**	**-**	**I89-T90**
**-**	**T90-S91**	**-**	**-**	**-**	**-**	**-**	**-**	**-**	**-**
**-**	**I94-Q95**	**-**	**-**	**-**	**-**	**-**	**I94-Q95**	**-**	**-**
**-**	**T96-A97**	**-**	**-**	**-**	**-**	**-**	**-**	**-**	**-**
**-**	**A97-V98**	**-**	**-**	**-**	**-**	**-**	**-**	**-**	**-**
**-**	**R99-L100**	**R99-L100**	**R99-L100**	**-**	**-**	**-**	**-**	**-**	**-**
**-**	**-**	**-**	**-**	**-**	**G104-E105**	**-**	**G104-E105**	**-**	**-**
**-**	**-**	**-**	**-**	**-**	**E105-L106**	**-**	**-**	**-**	**-**
**-**	**-**	**K108-H109**	**-**	**-**	**-**	**-**	**-**	**-**	**-**
**-**	**-**	**-**	**-**	**-**	**-**	**-**	**H109-A110**	**-**	**-**
**-**	**-**	**V111-S112**	**-**	**-**	**-**	**-**	**-**	**-**	**-**
**-**	**-**	**-**	**-**	**-**	**S112-E113**	**S112-E113**	**-**	**-**	**-**
**-**	**E113-G114**	**-**	**-**	**-**	**E113-G114**	**E113-G114**	**-**	**-**	**-**
**-**	**-**	**-**	**-**	**-**	**-**	**-**	**T115-K116**	**-**	**-**
**-**	**A117-V118**	**-**	**-**	**-**	**-**	**-**	**-**	**-**	**-**
**-**	**-**	**-**	**-**	**-**	**-**	**T119-K120**	**-**	**T119-K120**	**-**

* The molecular weights of the histone hydrolysis products were used for estimation of the corresponding sites of hydrolysis based on a set of data from 8–10 spectra. Missing splitting sites are marked with a dash (-).

**Table 3:** Sites of H2B histone hydrolysis by IgGs against five individual histones 20 days after mouse immunization with MOG and IgGs against MBP *.

Sites of H2A Hydrolysis by IgGs 20 Days after Mouse Immunization with MOG and Antibodies against MBP								
MOG20- aH1	MOG20- aH2A	MOG20- aH2B	MOG20- aH3	MOG20- aH4	Con- aMBP	MOG20- aMBP	DNA20- aMBP	DNA60- aMBP
26 Sites	26 Sites	7 Sites	6 Sites	11 Sites	11 Sites	14 Sites	9 Sites	4 Sites
K11-K12	K11-K12				-			
-	K16-A17	-	-	-	-	-	-	-
T19-K20	-	-	-	-	-	-	-	-
K20-A21	K20-A21	-	-	-	K20-A21	-	-	-
-	-	-	-	-	-	-	A21-Q22	-
-	K24-D25	K24-D25	-	-	K24-D25	K24-D25	-	-
-	-	-	-	-	-	K38-R29	-	-
-	K30-R31	-	-	-	-	-	-	-
R31-S32	R31-S32	-	-	R31-S32	-	-	-	-
E35-S36	-	E35-S36	-	-	-	-	-	-
	S36-Y37	-	-	-	S36-Y37	-	-	-
Y37-S38	-	-	-	Y37-S38	-	-	-	-
-	S38-I39	-	-	S38-I39	S38-I39	-	-	-
-	-	Y40-V41	-	Y40-V41	-	-	-	-
-	-	-	-	V41-Y42	V41-Y42	-	-	-
-	-	Y42-K43	Y42-K43	Y42-K43	-	Y42-K43	-	-
-	-	-	-	-	-	-	K43-V44	-
-	-	-	-	V44-L45	V44-L45	-	-	-
-	-	-	-	-	-	-	K46-Q47	
	Q47-V48	Q47-V48		Q47-V48	-	-	-	-
-	-	-	-	-	-	H49-P50	-	-
D51-T52	-	-	-	-	-	-	-	-
-	G53-I54	-	-	-	G53-I54	-	-	-
I54-S55	I54-S55	-	-	-	-	-	-	-
-	-	-	-	-	-	S55-S56	-	-
S56-K57	-	-	-	-	-	-	-	-
-	-	-	-	-	-	-		
M59-G60	-	-	-	-	-	M59-G60	-	-
-	-	-	-	-	-	-	-	-
-	G60-I61	-	-	-	-	-	-	-
-	I61-M62	-	-	-	-	-	-	-
-	-	-	-	-	-	-	-	-
-	F65-V66	-	-	-	-	-	-	-
-	I69-F70	-	I69-F70	-	I69-F70	-	-	-
E71-R72	E71-R72	-	-	-	-	-	-	-
-	-	-	-	-	-	R72-I73	-	-
-	I73-A74	-	I73-A74	-	-	-	-	-
-	-	-	-	-	-	A74-G75	A74-G75	-
-	E76-A77		E76-A77		E76-A77	E76-A77	E76-A77	-
-	-	-	-	-	-	A77-S78	A77-S78	-
-	-	-	-	-	-	S78-R79	S78-R79	-
R79-L80	-	-	-	R79-L80	-	-	-	-
-	-	-	-	-	-	A81-H82	-	A81-H82
-	-	-	-	-	-	Y83-N84	-	Y83-N84
N84-K85	-	-	-	-	-	-	-	-
-	K85-R86	-	-	-	-	-	-	-
R86-S87	R86-S87	-	-	R86-S87	R86-S87	-	R86-S87	-
-		S87-T88	S87-T88	-	-	-	-	-
T90-S91	T90-S91	-	-	-	-	-	-	-
T96-A97	T96-A97	-	-	-	-	-	T96-A97	-
-	V98-R99	-	-	-	-	-	-	-
R99-L100	-	-	-	-	-	R99-L100	-	-
L102-P103	-	-	-	-	-	-	-	-
G104-E105	-	-	-	-	-	-	-	-
E105-L106	-	-	-	-	-	-	-	-
V111-S112	-	-	-	-	-	-	-	-
S112-E113	-	-	-	-	-	-	-	-
E113-G114	-	-	-	-	-	-	-	-
	-	-	-	-	-	-	-	G114-G115
K116-A117	K116-A117	-	-	-	K116-A117	-	-	
K120-Y121	K120-Y121	-	-	-	-	-	-	-
Y121-T122	Y121-T122	Y121-T122	Y121-T122	Y121-T122	-	-	-	Y121-T122

* The molecular weights of the histone hydrolysis products were used for estimation of the corresponding sites of hydrolysis based on a set of data from 8–10 spectra. Missing splitting sites are marked with a dash (-).

**Table 4:** Sites of H2B histone hydrolysis by IgGs against five individual histones 20 and 60 days after mouse immunization with DNA-histone complex *.

Sites of H2B Hydrolysis with IgGs 20 and 60 Days after Mouse Immunization with DNA–Histone Complex									
DNA20- aH1	DNA60- aH1	DNA20- aH2A	DNA60- aH2A	DNA20- aH2B	DNA60- aH2B	DNA20- aH3	DNA60- aH3	DNA20- aH4	DNA60- aH4
7 Sites	8 Sites	10 Sites	5 Sites	15 Sites	33 Sites	3 Sites	11 Sites	10 Sites	6 Sites
**-**	**-**	**-**	**-**	**-**	**-**	**-**	**-**	**G13-S14**	**-**
**-**	**-**	**-**	**-**	**-**	**K20-A21**	**-**	**-**	**-**	**-**
**-**	**-**	**K24-D25**	**-**	**-**	**-**	**-**	**-**	**-**	**-**
**-**	**-**	**K28-R29**	**-**	**-**	**-**	**-**	**-**	**-**	**-**
**-**	**-**	**-**	**K30-R31**	**-**	**-**	**-**	**-**	**-**	**-**
**-**	**-**	**R31-S32**	**R31-S32**	**R31-S32**	**R31-S32**	**-**	**R31-S32**	**-**	**-**
**-**	**-**	**-**	**-**	**-**	**E35-S36**	**-**	**E35-S36**	**E35-S36**	**-**
**-**	**-**	**-**	**-**	**-**	**-**	**-**	**-**	**S36-Y37**	**-**
**-**	**Y37-S38**	**Y37-S38**	**Y37-S38**	**Y37-S38**	**Y37-S38**	**Y37-S38**	**-**	**-**	**-**
**-**	**-**	**-**	**-**	**S38-I39**	**-**	**S38-I39**	**-**	**-**	**-**
**-**	**-**	**Y40-V41**	**-**	**-**	**Y40-V41**	**-**	**-**	**-**	**-**
**-**	**Y42-K43**	**Y42-K43**	**-**	**Y42-K43**	**Y42-K43**	**Y42-K43**	**Y42-K43**	**Y42-K43**	**-**
**-**	**-**	**-**	**-**	**K43-V44**	**-**	**-**	**-**	**-**	**-**
**-**	**-**	**-**	**-**	**-**	**-**	**-**	**-**	**L45-K46**	**-**
**-**	**Q47-V48**	**-**	**-**	**-**	**-**	**-**	**-**	**-**	**-**
**H49-P50**	**-**	**-**	**-**	**-**	**H49-P50**	**-**	**-**	**-**	**-**
**-**	**-**	**-**	**-**	**-**	**D51-T52**	**-**	**-**	**-**	**-**
**-**	**-**	**-**	**-**	**I54-S55**	**I54-S55**	**-**	**I54-S55**	**-**	**-**
**-**	**-**	**-**	**-**	**-**	**S56-K57**	**-**	**-**	**S56-K57**	**-**
**-**	**-**	**-**	**-**	**K57-A58**		**-**	**-**	**-**	**-**
**-**	**-**	**-**	**-**	**M59-G60**	**M59-G60**	**-**	**M59-G60**	**-**	**-**
**-**	**-**	**-**	**-**	**-**	**G60-I61**	**-**	**-**	**-**	**-**
**-**	**-**	**-**	**-**	**-**	**M62-N63**	**-**	**-**	**-**	**-**
**-**	**-**	**-**	**-**	**-**	**F70-E71**	**-**	**-**	**-**	**-**
**-**	**-**	**-**	**-**	**-**	**E71-R72**	**-**	**E71-R72**	**E71-R72**	
**-**	**-**	**-**	**-**	**-**	**A74-G75**	**-**	**-**	**-**	**R72-I73**
**G75-E76**	**-**	**-**	**-**	**-**	**-**	**-**	**-**	**-**	**-**
**E76-A77**	**-**	**E76-A77**	**-**	**-**	**-**	**-**	**-**	**E76-A77**	**E76-A77**
**A77-S78**	**-**	**A77-S78**	**-**	**-**	**-**	**-**	**-**	**-**	**A77-S78**
**S78-R79**	**-**	**-**	**-**	**-**	**S78-R79**	**-**	**-**	**-**	**S78-R79**
**-**	**-**	**-**	**R79-L80**	**R79-L80**	**R79-L80**	**-**	**-**	**-**	**-**
**-**	**-**	**-**	**-**	**-**	**L80-A81**	**-**	**-**	**-**	**-**
**-**	**A81-H82**	**-**	**-**	**-**	**A81-H82**	**-**	**-**	**-**	**-**
**-**	**-**	**-**	**-**	**-**	**-**	**-**	**-**	**-**	**-**
**-**	**Y83-N84**	**-**	**-**	**-**	**Y83-N84**	**-**	**-**	**-**	**H82-Y83**
**-**	**N84-K85**	**-**	**-**	**N84-K85**	**-**	**-**	**N84-K85**	**-**	**-**
**-**	**-**	**-**	**-**	**-**	**K85-R86**	**-**	**-**	**-**	**-**
**R86-S87**	**R86-S87**	**-**	**R86-S87**	**R86-S87**	**R86-S87**	**-**	**-**	**-**	**R86-S87**
**-**	**-**	**-**	**-**	**-**	**T90-S91**	**-**	**-**	**-**	**-**
**-**	**-**	**-**	**-**	**-**	**R92-E93**	**-**	**-**	**-**	**-**
**-**	**-**	**-**	**-**	**-**	**T96-A97**	**-**	**T96-A97**	**-**	**-**
**-**	**-**	**V98-R99**	**-**	**V98-R99**	**-**	**-**	**-**	**-**	**-**
**-**	**-**	**-**	**-**	**-**	**R99-L100**	**-**	**R99-L100**	**-**	**-**
**-**	**-**	**-**	**-**	**L100-L101**	**L100-L101**	**-**	**-**	**-**	**-**
**-**	**-**	**-**	**-**	**-**	**G104-E105**	**-**	**G104-E105**	**-**	**-**
**-**	**-**	**-**	**-**	**K108-H109**	**-**	**-**	**-**	**-**	**-**
**-**	**-**	**-**	**-**	**-**	**-**	**-**	**V111-S112**	**-**	**-**
**-**	**-**	**-**	**-**	**-**	**S112-E113**	**-**	**-**	**S112-E113**	**-**
**-**	**-**	**-**	**-**	**-**	**E113-G114**	**-**	**-**	**-**	**-**
**-**	**-**	**-**	**-**	**-**	**116K-117A**	**-**	**-**	**-**	**-**
**Y121-T122**	**-**	**-**	**-**	**-**	**-**	**-**	**-**	**-**	**-**
**H49-P50**	**-**	**-**	**-**	**-**	**-**	**-**	**-**	**-**	**-**
**G75-E76**	**-**	**-**	**-**	**-**	**-**	**-**	**-**	**-**	**-**
**E76-A77**	**-**	**-**	**-**	**-**	**-**	**-**	**-**	**-**	**-**
**-**	**K120-Y121**	**-**	**-**	**-**	**-**	**-**	**-**	**-**	**-**
**-**	**-**	**Y121-T122**	**-**	**Y121-T122**	**Y121-T122**	**-**	**-**	**Y121-T122**	**-**

* The molecular weights of the histone hydrolysis products were used for estimation of the corresponding sites of t hydrolysis based on a set of data from 8–10 spectra. Missing splitting sites are marked with a dash (-).

## Data Availability

The data that supports the results of this study are included in the article and its supplementary material.

## Supplementary Materials

The following supporting information can be downloaded at: https://www.mdpi.com/article/10.3390/molecules28072973/s1, Supplementary data: Supplementary Figures S1–S3 demonstrate a change in different parameters during the development of EAE by EAE-prone mice. References [10,12,13] are cited in the supplementary materials.

## Abbreviations

Abs, antibodies; AGDs, antigenic determinants; Abzs, abzymes; AA, amino acid; AIDs, autoimmune diseases; BFU-E, erythroid burst-forming unit (early erythroid colonies); CFU-E, erythroid burst-forming unit (late erythroid colonies); CFU-GM, granulocyte-macrophage colony-forming units; CFU-GEMM, granulocyte-erythroid-megakaryocyte-macrophage colony-forming units; HIV-1, human immunodeficiency virus type 1; MBP, myelin basic protein; MS, multiple sclerosis; MALDI-TOF, matrix-assisted laser ionization/desorption time-of-flight mass spectrometry; SLE, systemic lupus erythematosus.
